# The Hippo kinase LATS2 impairs pancreatic β-cell survival in diabetes through the mTORC1-autophagy axis

**DOI:** 10.1038/s41467-021-25145-x

**Published:** 2021-08-13

**Authors:** Ting Yuan, Karthika Annamalai, Shruti Naik, Blaz Lupse, Shirin Geravandi, Anasua Pal, Aleksandra Dobrowolski, Jaee Ghawali, Marina Ruhlandt, Kanaka Durga Devi Gorrepati, Zahra Azizi, Dae-Sik Lim, Kathrin Maedler, Amin Ardestani

**Affiliations:** 1 Centre for Biomolecular Interactions Bremen, University of Bremen, Bremen, Germany; 2 Department of Molecular Medicine, School of Advanced Technologies in Medicine, Tehran University of Medical Sciences, Tehran, Iran; 3 Department of Biological Sciences, KAIST 291 Daehak-ro, Yuseong-gu, Daejeon, Republic of Korea; 4 Present Address: Institute of Cardiovascular Regeneration, Centre for Molecular Medicine, Goethe University Frankfurt, Frankfurt, Germany

**Keywords:** Cell biology, Cell death, Apoptosis, Endocrinology, Endocrine system and metabolic diseases

## Abstract

Diabetes results from a decline in functional pancreatic β-cells, but the molecular mechanisms underlying the pathological β-cell failure are poorly understood. Here we report that large-tumor suppressor 2 (LATS2), a core component of the Hippo signaling pathway, is activated under diabetic conditions and induces β-cell apoptosis and impaired function. LATS2 deficiency in β-cells and primary isolated human islets as well as β-cell specific LATS2 ablation in mice improves β-cell viability, insulin secretion and β-cell mass and ameliorates diabetes development. LATS2 activates mechanistic target of rapamycin complex 1 (mTORC1), a physiological suppressor of autophagy, in β-cells and genetic and pharmacological inhibition of mTORC1 counteracts the pro-apoptotic action of activated LATS2. We further show a direct interplay between Hippo and autophagy, in which LATS2 is an autophagy substrate. On the other hand, LATS2 regulates β-cell apoptosis triggered by impaired autophagy suggesting an existence of a stress-sensitive multicomponent cellular loop coordinating β-cell compensation and survival. Our data reveal an important role for LATS2 in pancreatic β-cell turnover and suggest LATS2 as a potential therapeutic target to improve pancreatic β-cell survival and function in diabetes.

## Abstract (web summary)

Diabetes is characterized by dysfunction and loss of beta-cells, and promoting beta-cell survival is of therapeutic interest. Here the authors show that Large-tumor suppressor 2 (LATS2), a core component of the Hippo signaling pathway, induces beta-cell failure through mTORC1 hyperactivation and autophagic flux suppression.

## Introduction

Both type 1 diabetes (T1D) and type 2 diabetes (T2D) result from an absolute or relative decline in pancreatic β-cell function and/or mass^1, 2^, and β-cell apoptosis is its hallmark^3–6^. T1D is an autoimmune disease resulting from selective destruction of pancreatic islet β-cells^7^. T2D is a complex metabolic disorder characterized by insulin resistance as well as decreased insulin secretory function and ultimately reduced β-cell mass, resulting in the development of chronic β-cell dysfunction and relative insulin deficiency^8^. Given these complex causes of β-cell failure, inhibition of apoptosis and/or β-cell dysfunction represents a potential therapeutic intervention to the treatment of diabetes. Understanding the varied β-cell responses to metabolic assaults and how diabetes-related signals impair β-cell function and survival is important for the understanding of pathogenesis as well as target identification towards a β-cell-directed therapy of diabetes.

Hippo signaling—first discovered using genetic screens in Drosophila—is an evolutionarily conserved pathway that critically regulates development, growth, and homeostasis of various tissues in response to a wide range of extra- and intracellular signals^9^. The mammalian Hippo pathway constitutes core kinases (MST1/2 and LATS1/2), adaptor proteins (SAV1 for MST1/2 and MOB1 for LATS1/2), downstream terminal effectors (YAP and TAZ), and transcription factors (TEAD1-4). Mammalian Sterile 20-like kinases (MST1/2) and Large-tumor suppressors (LATS1/2) represent core kinases of the Hippo pathway. MST1/2, in complex with a regulatory protein Salvador (Sav1), phosphorylates and activates LATS1/2 kinases, which also form a complex with a regulatory protein Mps-one binder 1 (MOB1). The function of the effector transcriptional coactivator Yes-associated protein (YAP) is therefore mainly regulated by phosphorylation-dependent mechanisms. The kinases LATS1/2 inactivate YAP by direct phosphorylation on multiple serine residues including at S127, enhancing YAP binding to 14-3-3 proteins, its cytoplasmic sequestration and subsequent proteasomal degradation. In association with TEA domain (TEAD) family transcription factors, YAP fosters the expression of target genes, with pro-proliferative and anti-apoptotic outcomes^9–12^.

Hippo’s dysregulation has been implicated in many human disorders such as cancer and metabolic diseases^9, 13^. Hippo components such as MST1, Merlin/NF2, YAP, and TEAD control various aspects of β-cell life including development, β-cell function, survival, and proliferation^13–20^. For example, MST1 is an important regulator of pancreatic β-cell death and dysfunction in human and rodent β-cells. MST1 inhibition restores normoglycemia, β-cell function, and survival under diabetic conditions in vitro and in vivo^14, 21^. LATS2, an MST1 downstream substrate, is a ubiquitously expressed serine/threonine kinase and involved in multiple cellular processes such as morphogenesis, proliferation, stress responses, apoptosis, and differentiation^22–27^. LATS2 promotes cell death through regulation of multiple downstream targets such as P53, FOXO1, c-Abl, and YAP^24, 28–31^. In this regard, the MST1-LATS2 axis is an important regulator of apoptosis in the heart: knockdown or genetic deletion of MST1 or LATS2 in cardiomyocytes provides protection against ischemic injury^26, 30, 32^. As YAP is excluded from mature β-cells during development^13–19, 33^, MST1 and LATS2 core kinases can also act independently of their classical terminal effector YAP. In this study, we investigated whether LATS2 hyper-activation would trigger β-cell death and impaired insulin secretion, whether its deficiency would promote β-cell survival under diabetic conditions in vitro and in vivo as well as the molecular mechanism of pathogenic action of LATS2 in the β-cells.

## Results

### LATS2 was activated under diabetogenic conditions and induced β-cell death and dysfunction

**Fig. 1:**
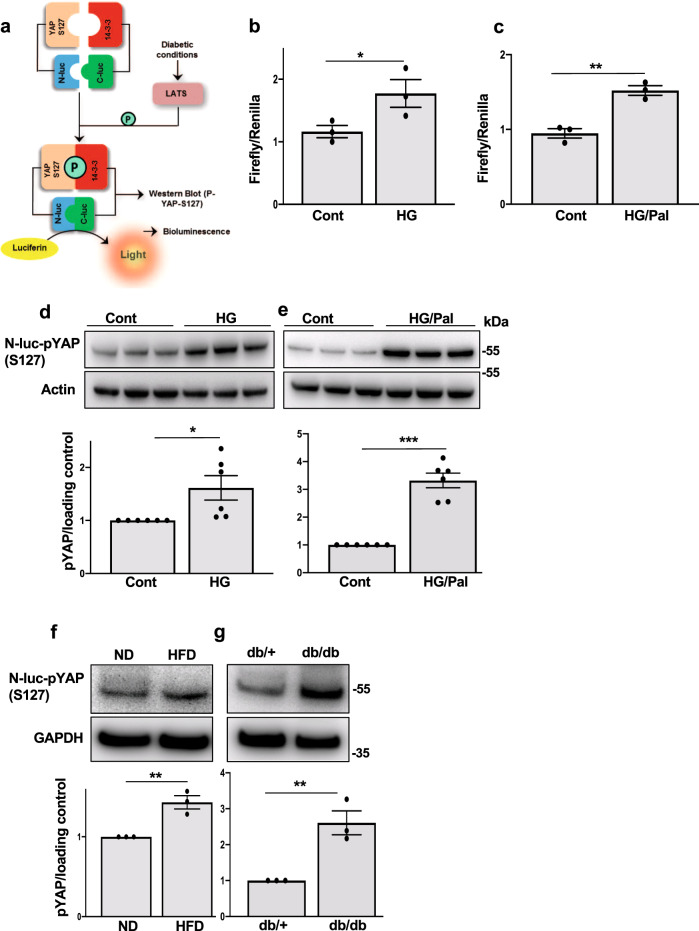
LATS2 is activated under diabetogenic conditions. **a** Schematic structure and mechanism of action for LATS-BS. **b**–**e** INS-1E cells transfected with the firefly luciferase reporter plasmids N-luc-YAP15-S127, C-luc-14-3-3, and pRL-Renilla luciferase were treated with 22.2 mM glucose alone or in combination with 0.5 mM palmitate for 24 h. **b**, **c** Downstream phosphorylation of the exogenously expressed YAP-S127 fragment was determined by firefly luciferase activity and normalized to the Renilla signal (*n* = 3 biologically independent samples). **d**, **e** Representative western blots and pooled quantitative densitometry analysis for exogenously expressed YAP-127 by a phospho-specific antibody are shown (*n* = 6 biologically independent samples). Isolated islets from **f** HFD-treated C57BL/6 J mice for 16 weeks or **g** the obese diabetic leptin receptor-deficient db/db mice and their corresponding controls cultured overnight and transfected with the N-luc-YAP15-S127 plasmid for 24 h. Representative western blots and pooled quantitative densitometry are shown (*n* = 3 independent experiments) and results were normalized to the respective control conditions and ratios, in which a normal distribution of results cannot be proven, were analyzed. Data are expressed as means ± SEM. *p < 0.05, **p < 0.01, ***p < 0.001; all by two-tailed Student’s *t-*tests.

In order to identify whether LATS is activated in response to diabetogenic stimuli, a recently established bioluminescence-based biosensor (LATS-BS) that monitors the activity of LATS kinase in cells in real-time with accurate quantification, high sensitivity, and excellent reproducibility has been used^34^ (Fig. 1a). LATS1/2 kinases phosphorylate their own established target YAP on S127, which exposes the docking site for binding of 14-3-3 proteins leading to YAP cytoplasmic sequestration. LATS-BS consists of a minimal YAP fragment that interacts with 14-3-3 in a phosphorylation-dependent manner. Therefore, a LATS-BS construct has been generated with fusion of YAP fragment and 14-3-3 with N-terminal and C-terminal Firefly luciferase fragments (N-luc and C-luc), respectively that measures LATS kinase activity by assessing the complementation between pS127-YAP and 14-3-3 in a LATS-phosphorylation-dependent manner^34^ (Fig. 1a). The β-cell line INS-1E was transfected with LATS-BS and control Renilla constructs and then exposed chronically to increased glucose concentrations (glucotoxicity), or its combination with the free fatty acid palmitate (glucolipotoxicity). Both diabetogenic treatments increased bioluminescence signals indicating enhanced exogenously expressed YAP phosphorylation at S127 (LATS specific site) and thus hyper-activated Hippo kinases LATS1/2 (Fig. 1b, c). LATS1/2 hyper-activation under diabetic conditions was further confirmed by immunoblot analysis of increased exogenously expressed YAP-S127 phosphorylation levels triggered by high glucose as well as high glucose/palmitate (Fig. 1d, e). Since the Hippo pathway adaptor protein MOB1 interacts with and activates LATS1/2 kinase activity^23, 35^, we analyzed the protein expression of MOB1 in β-cells. Prolonged culture of INS-1E cells under high glucose or high glucose/palmitate up-regulated MOB1 protein levels (Supplementary Fig. 1a). Also, overexpression of LATS2 itself increased MOB1 levels in INS-1E β-cells and human islets (Supplementary Fig. 1b, c). LATS activation (as represented by exogenously expressed pYAP levels) and MOB1 were also increased in islets of hyperglycemic high fat/ high sucrose (HFD) fed mice for 16 weeks (Fig. 1f and Supplementary Fig. 1d). Similarly, exogenously expressed pYAP and MOB1 protein levels were robustly elevated in islets of another model of T2D, the obese diabetic leptin receptor-deficient db/db mice (Fig. 1g and Supplementary Fig. 1e). These data show that LATS activity and its associated protein MOB1 is markedly elevated by pro-diabetic conditions in metabolically stressed β-cells and islets isolated from mouse models of diabetes.

**Fig. 2:**
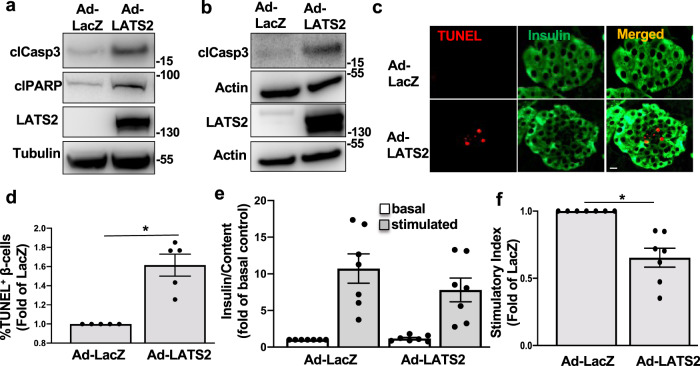
LATS2 induces β-cell death and dysfunction. INS-1E cells (**a**) and human islets (**b**–**f**) transduced with LacZ control or LATS2 adenoviruses for 48 h. **a**, **b** Representative western blots, **c** double staining for TUNEL (red) and insulin (green) and **d** pooled TUNEL analysis (**c**, **d**: *n* = 5 different human islets isolations). **e** Insulin secretion during 1 h incubation with 2.8 mM (basal) and 16.7 mM (stimulated) glucose, normalized to insulin content. **f** Insulin stimulatory index denotes the ratio of stimulated (16.7 mM glucoses) and basal (2.8 mM glucose) (**e**, **f**: *n* = 7 different human islets isolations). All western blots show representative results from at least three independent experiments. Data are expressed as means ± SEM. *p < 0.001; all by two-tailed Student’s *t-*tests. scale bar depicts 10 μm.

In subsequent experiments we investigated whether LATS2 overexpression is directly detrimental for β-cell survival. LATS2 was overexpressed through adenoviral transduction, which efficiently up-regulated LATS2 (Fig. 2a, b). Overexpression of LATS2 itself was sufficient to induce β-cell apoptosis in both INS-1E cells and human islets, as determined by cleavage of caspase-3 and poly-(ADP-ribose) polymerase (PARP), a downstream substrate of caspase 3 (Fig. 2a, b). In line with these findings, LATS2 overexpression increased the number of TUNEL-positive apoptotic β-cells in human islets confirming β-cell-specific induction of apoptosis by LATS2 hyper-activation (Fig. 2c, d). Furthermore, in isolated human islets, LATS2 overexpression led to impairment of glucose-stimulated insulin secretion (GSIS; Fig. 2e, f). Together, our data show that LATS2 impairs β-cell survival and function.

### Loss of the LATS2-MOB1 axis improved β-cell survival in vitro

**Fig. 3:**
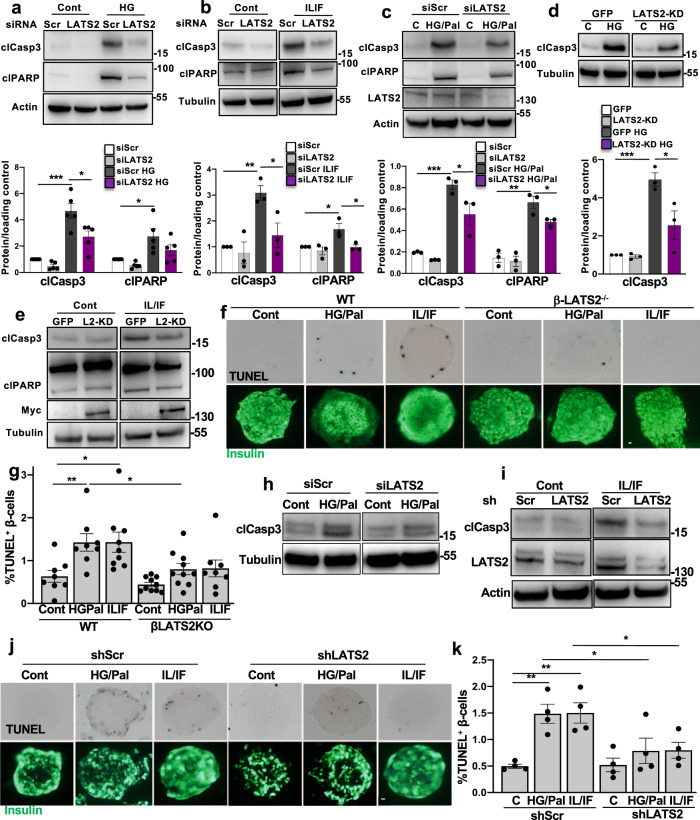
Loss of the LATS2 improves β-cell survival in vitro. **a**–**c** Representative western blots and pooled quantitative densitometry analysis (lower panels) of INS-1E cells transfected with LATS2 siRNA or control siScr and treated with **a** 22.2 mM glucose, **b** 2 ng/mL IL1β (IL) plus 1000 U/mL IFNγ (IF) and **c** 22.2 mM glucose plus 0.5 mM palmitate for 48 h (*n* = 5, 3 and 3 independent experiments, respectively for **a**–**c**). **d**, **e** Representative western blots and pooled quantitative densitometry analysis (lower panel) of INS-1E cells transfected with GFP or kinase-dead form of LATS2 (LATS2-KD) and then treated with **d** 22.2 mM glucose or **e** mixture of IL/IF for 48 h (*n* = 3 independent experiments). **f**, **g** Isolated islets from β-LATS2KO and Rip-Cre control mice were recovered after isolation overnight and exposed to diabetogenic conditions (IL-1β/IFNγ or the mixture of 22.2 mM glucose and 0.5 mM palmitate (HG/Pal)) for 72 h. β-cell apoptosis was analyzed by double staining of TUNEL (black nuclei), and insulin (green). Representative images (**f**) and quantitative percentage of TUNEL positive β-cells (**g**) are shown (*n* = 8, 8, 9, 10, 10, 8 mice, respectively for WT cont, WT HG/Pal, WT IL/IF, β-LATS2-KO cont, β-LATS2-KO HG/Pal, and β-LATS2-KO IL/IF conditions). **h**, **i** Representative western blots of human islets transfected with siLATS2 or transduced with Ad-hShLATS2 and treated with **h** 22.2 mM glucose plus 0.5 mM palmitate or **i** mixture of IL/IF for 72 h (*n* = 2 different human islets isolations). **j**, **k** Human islets transduced with Ad-hShLATS2 or Ad-shScr control were exposed to diabetogenic conditions (IL-1β/IFNγ or the mixture of 22.2 mM glucose and 0.5 mM palmitate (HG/Pal)) for 72 h. β-cell apoptosis was analyzed by double staining of TUNEL (black nuclei) and insulin (green). Representative images (**j**) and quantitative percentage of TUNEL positive β-cells (**k**) (*n* = 4 different human islets isolations) are shown. All lanes were run on the same gel but were noncontiguous (**b**, **d**, **e**, **h**, **i**). Data are expressed as means ± SEM. Pooled quantitative densitometry of western blots were normalized to the respective control conditions and ratios (except c), in which a normal distribution of results cannot be proven, were analyzed. *p < 0.05, **p < 0.01, ***p < 0.001; all by two-tailed Student’s *t-*tests. scale bar depicts 10 μm.

Further analyses aimed to investigate whether LATS2 inhibition can rescue β-cells from apoptosis under diabetogenic conditions. β-cells were transfected with small interfering RNA (siRNA) against LATS1 and/or LATS2 and then exposed to glucotoxic and glucolipotoxic conditions as well as the mixture of pro-inflammatory cytokines interleukin-1beta (IL-1β) and interferon-gamma (IFN-γ). Depletion of endogenous LATS2 but not LATS1 activity protected β-cells from glucose-, glucose/palmitate- and cytokine-induced apoptosis as demonstrated by decreased caspase-3- and PARP-cleavage (Fig. 3a–c and Supplementary Fig. 2a, b) showing a potential functional diversity between LATS1 and LATS2 in the regulation of β-cell apoptosis. The pro-survival function of loss of LATS2 in the β-cells was further validated by a second siRNA pool to the LATS2 gene with comparable gene silencing efficiency (Supplementary Fig. 2c). LATS kinases redundantly exert phosphorylation-induced inactivation of the transcription factors YAP/TAZ^36^. We thus assessed whether LATS2’s canonical kinase activity was necessary for its pro-apoptotic function by overexpressing LATS2 kinase dead (KD) mutant (K697R), where the ATP-binding site K697 was mutated to another positively charged amino acid arginine (R)^37^. LATS2-KD overexpression profoundly reduced the levels of β-cell apoptosis under diabetogenic conditions (Fig. 3d, e). Also, β-cell specific LATS2 deletion in mice markedly suppressed the number of TUNEL-positive β-cells under a pro-diabetic milieu in vitro (Fig. 3f, g). In human islets, LATS2 was silenced by siRNA-mediated transfection as well as adenoviral-mediated infection either with Ad-shLATS2 or control shScr viruses. In line with rodent β-cells, apoptosis triggered by pro-inflammatory cytokines as well as by the mixture of high glucose/palmitate was diminished by LATS2 knockdown in isolated primary human islets (Fig. 3h, i). Consistently, repression of LATS2 by shRNA-mediated depletion of LATS2 expression efficiently prevented human β-cell death induced by diabetogenic conditions in human islets (Fig. 3j, k). Our data show that loss of LATS2 expression markedly protected both rodents and human β-cells from apoptosis under several diabetic conditions in vitro.

**Fig. 4:**
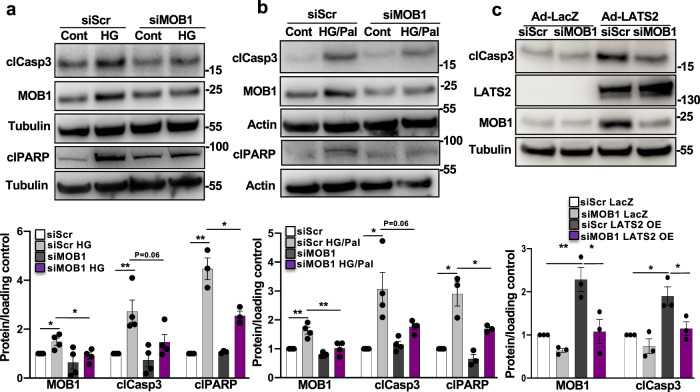
MOB1 knockdown protects from β-cell apoptosis in vitro. **a**, **b** Representative western blots and pooled quantitative densitometry analysis (lower panels) of INS-1E cells transfected with siMOB1 or control siScr and treated with **a** 22.2 mM glucose or **b** 22.2 mM glucose plus 0.5 mM palmitate for 48 h (*n* = 4 independent experiments; *n* = 3 for clPARP). **c** Representative western blot and pooled quantitative densitometry analysis (lower panel) of INS-1E cells transfected with siMOB1 or siScr and transduced with Ad-LacZ or Ad-LATS2 for 48 h (*n* = 3 independent experiments). Results were normalized to the respective control conditions and ratios, in which a normal distribution of results cannot be proven, were analyzed. Data are expressed as means ± SEM. *p < 0.05, **p < 0.01; all by two-tailed Student’s *t-*tests.

Mechanistically, silencing of endogenous LATS2 abolished MOB1 levels induced by prolonged treatment with high glucose suggesting a LATS2-dependent regulation of MOB1 by pro-diabetic stimuli (Supplementary Fig. 2d). To further prove such regulatory LATS2-MOB1 axis, MOB1 was silenced in order to directly assess its pro-apoptotic function. Knockdown of MOB1 antagonized the apoptotic effect of high glucose as well as high glucose/palmitate in INS-1E β-cells (Fig. 4a, b). Consistently, MOB1 knockdown antagonized LATS2-induced caspase-3 cleavage (Fig. 4c) indicating an indispensable role of MOB1 in the mechanism of LATS2-induced β-cell apoptosis. These data suggest the LATS2-MOB1 axis as a determinant for β-cell apoptosis under a diabetic milieu in β-cells.

### β-cell specific LATS2 ablation protected from STZ induced diabetes in vivo

**Fig. 5:**
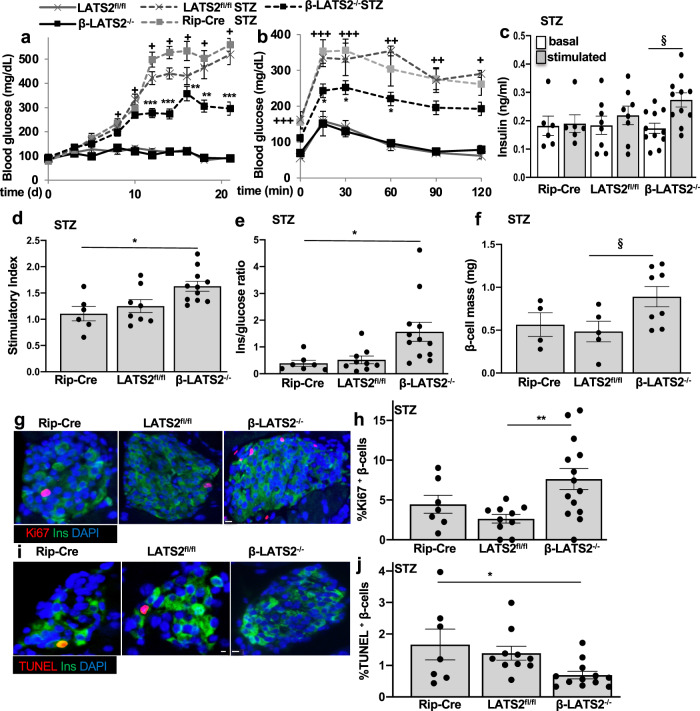
β-cell specific LATS2 ablation protects from STZ induced diabetes in vivo. **a**–**j** β-LATS2^−/−^ mice (*n* = 14), RIP-Cre (*n* = 7) and LATS2^fl/fl^ controls (*n* = 10) injected with streptozotocin (STZ) (40 mg per kg body weight for 5 consecutive days) or saline (β-LATS2^−/−^ *n* = 4; LATS2^fl/fl^ *n* = 5) for 5 consecutive days. **a** Random fed blood glucose measurements after first saline or STZ injection (day 0) over 21 days and **b** i.p. glucose tolerance test (GTT) at day 19 in β-LATS2^−*/*−^, Rip-Cre and LATS2^*fl/fl*^ mice. **c** Insulin secretion during an i.p.GTT measured before (0 min) and 15 min after glucose injection and expressed **d** as ratio of secreted insulin at 15 min to that secreted at 0 min (stimulatory index) (Rip-Cre STZ *n* = 6; LATS2^fl/fl^ STZ *n* = 8; β-LATS2^−/−^ STZ *n* = 11). **e** Ratio of secreted insulin and glucose calculated at fed state (Rip-Cre STZ *n* = 7; LATS2^fl/fl^ STZ *n* = 9; β-LATS2^−/−^ STZ *n* = 12). **f**–**j** Mice were sacrificed at day 22. **f** β-cell mass (given as percentage of the whole pancreatic section from 10 sections spanning the width of the pancreas) (Rip-Cre STZ *n* = 4; LATS2^fl/fl^ STZ *n* = 5; β-LATS2^−/−^ STZ *n* = 8). Representative images and quantitative analyses from triple staining for Ki67 (**g**, **h**) or TUNEL (**i**, **j**), insulin and DAPI expressed as percentage of TUNEL- or Ki67-positive β-cells ±SEM (Ki67: Rip-Cre STZ *n* = 7; LATS2^fl/fl^ STZ *n* = 10; β-LATS2^−/−^ STZ *n* = 14; TUNEL: Rip-Cre STZ *n* = 7; LATS2^fl/fl^ STZ *n* = 10; β-LATS2^−/−^ STZ *n* = 12). Data are expressed as means ± SEM. ^+^*p* < 0.001 LATS2^fl/fl^-STZ or RIP-Cre-STZ to LATS2^fl/fl^ control mice. ^++^*p* < 0.01 LATS2^fl/fl^-STZ or RIP-Cre-STZ to LATS2^fl/fl^ control mice. ^+++^*p* < 0.05 LATS2^fl/fl^-STZ or RIP-Cre-STZ to LATS2^fl/fl^ control mice. *p < 0.05 β-LATS2^−/−^-STZ to LATS2^fl/fl^-STZ or RIP-Cre-STZ. **p < 0.01 β-LATS2^−/−^-STZ to LATS2^fl/fl^-STZ or RIP-Cre-STZ. ***p < 0.001 β-LATS2^−/−^-STZ to LATS2^fl/fl^-STZ or RIP-Cre-STZ. ^§^*p* < 0.05. One-way ANOVA with Tukey’s post hoc test for (**a**, **b**, **d**, **e**, **h**, **j**); by two-tailed Student’s *t-*tests for (**c**, **f**). scale bar depicts 10 μm.

As LATS2 depletion protected from β-cell apoptosis under multiple diabetic conditions in vitro, we hypothesized that LATS2 deficiency may protect from diabetes development in vivo. To test this hypothesis, we generated β-cell-specific LATS2 knockout mice (β-LATS2^−/−^) by crossing LATS2 floxed (LATS2^*fl/fl*^) mice with the β-cell-specific Cre transgenic line driven by the rat insulin-2 promoter (Rip-Cre). Cre recombinase expression, as well as Rip-Cre-mediated specific deletion of LATS2 gene in pancreatic β-cell, was confirmed in isolated islets from β-LATS2^−/−^ and LATS2^*fl/fl*^ mice (Supplementary Fig. 3a). As Rip-Cre has been reported to delete genes in β-cells but also in hypothalamic neurons^38^, the specificity of LATS2 gene deletion was tested in genomic DNA from isolated liver, spleen, kidney, heart, hypothalamus, and pancreatic islets of β-LATS2^−/−^ mice. PCR analysis demonstrated that Cre-mediated LATS2 deletion was islet specific with no leakage in the hypothalamus or any other tested tissues (Supplementary Fig. 3b). β-LATS2^−/−^ mice were viable, fertile and showed no difference in food intake and body weight neither under a normal (ND) nor under a high fat diet (HFD) compared to LATS2^*fl/fl*^ mice, or to LoxP-negative mice (Supplementary Fig. 3c, d). To assess whether β-LATS2^−/−^ might protect against β-cell injury and diabetes, we induced diabetes by multiple-low dose streptozotocin (MLD-STZ) injection in β-LATS2^−/−^, LATS2^*fl/fl*^, and Rip-Cre mice. While MLD-STZ injection induced progressive hyperglycemia and severely impaired glucose tolerance in LATS2^*fl/fl*^ and Rip-Cre mice, random blood glucose levels were significantly reduced and glucose tolerance improved in β-LATS2^−/−^ mice (Fig. 5a, b). Also, glucose-induced insulin secretion was fully blunted in MLD-STZ-treated LATS2^*fl/fl*^ and Rip-Cre mice, but significantly restored in β-LATS2^−/−^ mice, together with an increased insulin-to-glucose ratio, compared to both LATS2^*fl/fl*^ and Rip-Cre controls (Fig. 5c–e). Histological examination of the pancreas and quantification of β-cell mass revealed an increased β-cell mass in STZ-injected β-LATS2^−/−^ mice, compared to control groups (Fig. 5f). To identify whether the restoration in β-cell mass was a result of increased β-cell numbers due to enhanced β-cell proliferation and/or decreased β-cell apoptosis, we next assessed β-cell proliferation and apoptosis in response to MLD-STZ treatment. A significant increase in double-labeled proliferation marker Ki67/insulin-positive β-cells was observed in MLD-STZ-treated β-LATS2^−/−^ relative to control from LATS2^*fl/fl*^ mice (Fig. 5g, h). Additionally, TUNEL-positive β-cells were markedly reduced in β-LATS2^−/−^ mice compared to the control group (Fig. 5i, j). This suggests a combined additive impact on augmented proliferation as well as reduced apoptosis as mechanism of β-cell mass restoration in β-LATS2^−/−^ mice. Altogether, our data show that β-cell-specific ablation of LATS2 diminished progressive hyperglycemia and improved glucose tolerance, insulin secretion, and β-cell mass in the MLD-STZ mouse model of β-cell destruction and diabetes.

### β-cell specific LATS2 ablation protected from HFD induced diabetes in vivo

**Fig. 6:**
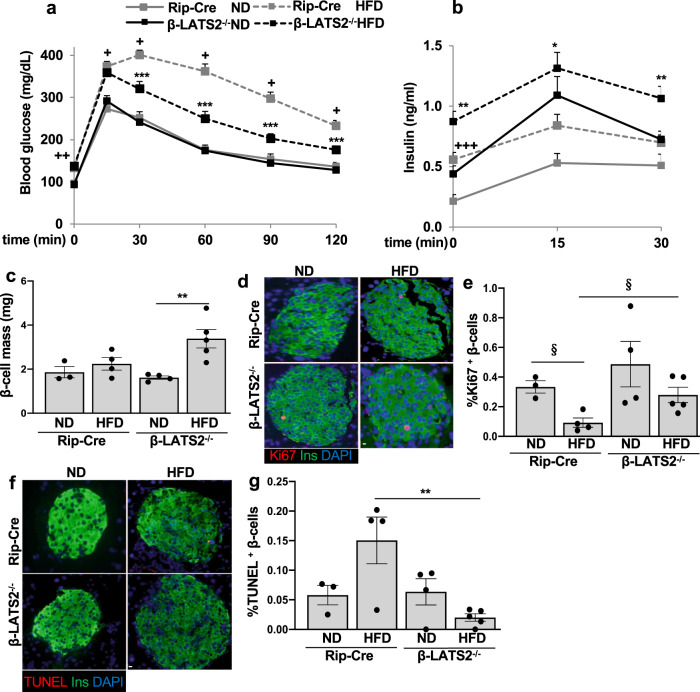
β-cell specific LATS2 ablation protects from HFD induced diabetes in vivo. **a**–**g** β-LATS2^−/−^ and Rip-Cre control mice were fed a normal (ND) or high fat/ high sucrose diet (“Surwit”; HFD) for 17 weeks. **a** intraperitoneal glucose tolerance test (ipGTT) (Rip-Cre ND *n* = 8; Rip-Cre HFD *n* = 21; β-LATS2^−/−^ ND *n* = 10; β-LATS2^−/−^ HFD *n* = 19). **b** Insulin secretion during an ipGTT measured before (0 min), 15 and 30 min after glucose injection (Rip-Cre ND *n* = 7; Rip-Cre HFD *n* = 18; β-LATS2^−/−^ ND *n* = 10; β-LATS2^−/−^ HFD *n* = 15). **c**–**g** Mice were sacrificed at week 17. **c** β-cell mass given as percentage of the whole pancreatic section from 10 sections spanning the width of the pancreas (Rip-Cre ND *n* = 3; Rip-Cre HFD *n* = 4; β-LATS2^−/−^ ND *n* = 4; β-LATS2^−/−^ HFD *n* = 5). Representative images and quantitative analyses from triple staining for Ki67 (**d**, **e**) or TUNEL (**f**, **g**), insulin and DAPI expressed as percentage of TUNEL- or Ki67-positive β-cells (Rip-Cre ND *n* = 3; Rip-Cre HFD *n* = 4; β-LATS2^−/−^ ND *n* = 4; β-LATS2^−/−^ HFD *n* = 5). Data are expressed as means ± SEM. ^+^*p* < 0.001 RIP-Cre-HFD to RIP-Cre ND mice. ^++^*p* < 0.01 RIP-Cre-HFD to RIP-Cre ND mice. ^+++^*p* < 0.05 RIP-Cre-HFD to RIP-Cre ND mice. *p < 0.05 β-LATS2^−/−^-HFD compared to RIP-Cre-HFD mice. **p < 0.01 β-LATS2^−/−^-HFD compared to RIP-Cre-HFD mice. ***p < 0.001 β-LATS2^−/−^-HFD compared to RIP-Cre-HFD mice. ^§^*p* < 0.05. All by one-way ANOVA with Tukey’s post hoc test except “**e**” by two-tailed Student’s *t-*tests. Scale bar depicts 10 μm.

In a second model of diet induced diabetes, we checked whether LATS2 is critical for the long-term β-cell compensatory response by subjecting 8-week-old control Rip-Cre and β-LATS2^−/−^ mice to normal or diabetogenic high-fat/high sucrose diets (ND or HFD) for 17 weeks, which led to chronic hyperglycemia, insulin resistance as well as β-cell failure in wild-type mice^14, 39^. HFD treatment revealed impaired glucose tolerance in Rip-Cre control mice compared to the ND-treated group. Conversely, HFD fed β-LATS2^−/−^ mice exhibited marked improvement in glucose tolerance relative to the HFD Rip-Cre control (Fig. 6a). To further examine β-cell function, an in vivo GSIS was performed. A significant increase in basal (0 min) and glucose-stimulated insulin levels (15 and 30 min) was found in HFD fed β-LATS2^−/−^ mice compared to Rip-Cre counterparts (Fig. 6b) indicating higher insulin secretion in LATS2 deleted β-cells. Consistent with the improved metabolic phenotype in the HFD model, a marked compensatory β-cell mass expansion in response to high-fat/high sucrose feeding was observed in the β-LATS2^−/−^ mice. In contrast, Rip-Cre control mice failed to compensatively increase β-cell mass in response to HFD feeding (Fig. 6c). We next analyzed β-cell proliferation and death in response to HFD. As expected, an increase in Ki-67 positive proliferating β-cells in the β-LATS2^−/−^ mice vs. their Rip-Cre controls was observed when HFD-treated animals were compared (Fig. 6d, e). While β-cell apoptosis remained unchanged in β-LATS2^−/−^ mice under the ND, HFD-treated β-LATS2^−/−^ mice showed a highly attenuated response; the number of TUNEL-positive β-cells was ∼70% reduced, compared to HFD-treated Rip-Cre control mice (Fig. 6f, g). This is consistent with the anti-apoptotic effect of LATS2 depletion observed in vitro and in the STZ in vivo model. The glucose-lowering effect of LATS2 deletion seems to be fully attributable to the β-cell, as β-LATS2^−/−^ HFD mice exhibited comparable insulin sensitivity to that of age-matched HFD-treated Rip-Cre control mice, irrespective of the diet (Supplementary Fig. 3e).

Taken together, these data suggest that the HFD induced LATS2 hyper-activation has a detrimental impact on β-cell viability, β-cell compensatory response and insulin secretion in the diet induced HFD model of β-cell decompensation and diabetes.

### LATS2 induced β-cell apoptosis by activating the mTORC1 pathway

**Fig. 7:**
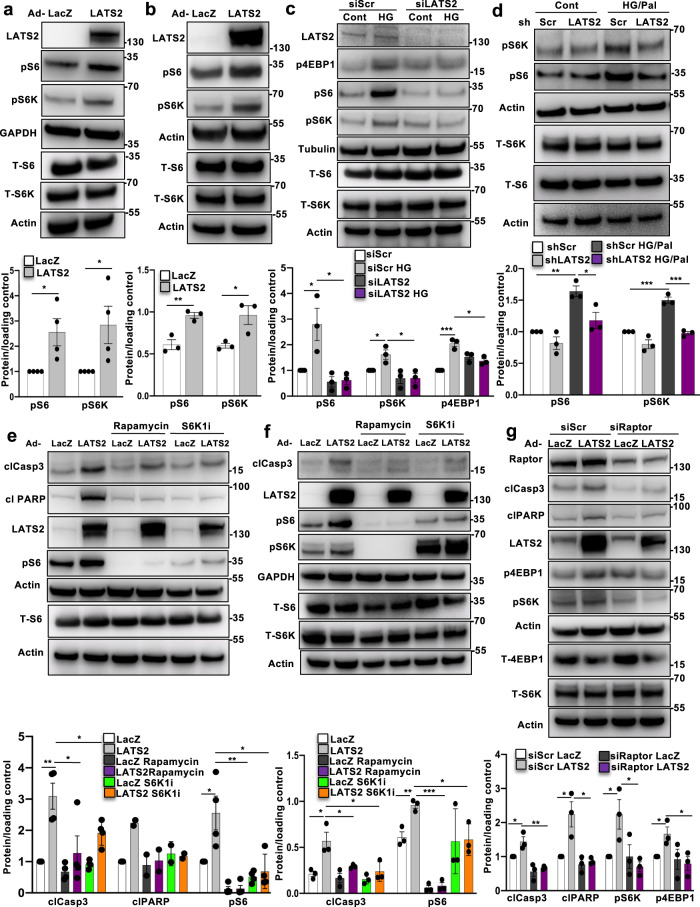
LATS2 induces β-cell apoptosis by activating mTORC1 pathway. **a**, **b** Representative western blots and pooled quantitative densitometry analysis (lower panels) of INS-1E cells (**a**) and human islets (**b**) transduced with LacZ control or LATS2 adenoviruses for 48 h (*n* = 4 and 3 independent experiments respectively for **a**, **b**). **c** Representative western blot and pooled quantitative densitometry analysis (lower panel) of INS-1E cells transfected with LATS2 siRNA or control siScr and treated with the 22.2 mM glucose (*n* = 3 independent experiments). **d** Representative western blot and pooled quantitative densitometry analysis (lower panel) of human islets transduced with Ad-hShLATS2 and treated with 22.2 mM glucose plus 0.5 mM palmitate for 72 h (*n* = 3 different human islets isolations). **e**, **f** Representative western blots and pooled quantitative densitometry analysis (lower panels) of INS-1E cells (**e**) and human islets (**f**) transduced with LacZ control or LATS2 adenoviruses for 24 h and then exposed to 100 nM Rapamycin or 10 μM S6K1 inhibitor (S6K1i) for additional 24 h (*n* = 4 and 3 independent experiments respectively for (**e**, **f**); *n* = 2 for clPARP). **g** Representative western blot and pooled quantitative densitometry analysis (lower panel) of INS-1E cells transfected with siRaptor or siScr and then transduced with Ad-LacZ or Ad-LATS2 for 48 h (*n* = 3 independent experiments). Data are expressed as means ± SEM. Pooled quantitative densitometry of western blots were normalized to the respective control conditions and ratios (except b, f), in which a normal distribution of results cannot be proven, were analyzed. *p < 0.05, **p < 0.01, ***p < 0.001; all by two-tailed Student’s *t-*tests.

Previous studies revealed β-cell-mTORC1 hyper-activation under chronic diabetogenic conditions; in islets isolated from T2D patients and from animal models of T2D as well as in β-cells cultured under diabetes-associated glucotoxic conditions^40–43^. It is still not well-understood which signals regulate such mTORC1 hyperactivation and downstream β-cell apoptosis. Both, Hippo and mTORC1 are major growth/viability regulating pathways in our cells and it is not surprising that they mutually control each other^44–47^. mTORC1 activity was profoundly elevated by LATS2 overexpression in INS-1E β-cells (Fig. 7a) and isolated human islets (Fig. 7b). Hyper-activation of mTORC1 was demonstrated by increased phosphorylation of its downstream target S6K1 at Thr 389 (pS6K), and the direct S6K substrate ribosomal protein S6 at Ser 235/236 (pS6) (Fig. 7a, b). Also, inhibition of mTORC1 by genetic and pharmacological tools restores insulin secretion in human T2D islets as well as in islets of diabetic mice^41, 48^ and corrects metabolic derangement in metabolically stressed β-cells^49^. Therefore, we further analyzed the LATS2-mTORC1 crosstalk and whether mTORC1 mediates the pro-apoptotic function of LATS2 in the context of diabetes. In order to define whether LATS2 regulate mTORC1 activity under diabetic conditions, LATS2 was first silenced and then, islets/β-cells were exposed to elevated glucose or its combination with palmitate. LATS2 knockdown resulted in decreased levels of pS6K1, pS6, and p4EBP1 (mTORC1 readouts) which were up-regulated upon exposure to a diabetic milieu in INS-1E β-cells and human islets providing a direct evidence for mTORC1 regulation by LATS2 (Fig. 7c, d). To establish whether inhibition of mTORC1-S6K1 signaling is sufficient to block pro-apoptotic function of LATS2 in β-cells, we blocked mTORC1 by the use of selective inhibitors against mTORC1 (rapamycin) and S6K1 (PF-4708671; S6K1i)^50^ (Fig. 7e, f). Rapamycin and S6K1i fully blocked LATS2-induced mTORC1 (represented by pS6) in INS-1E β-cells and human islets; together with counteracting apoptosis, as observed by reduced caspase-3 or PARP cleavage (Fig. 7e, f). In line with this observation, selective inhibition of endogenous mTORC1 by siRNA-mediated silencing of Raptor, mTORC1’s critical subunit, efficiently reduced mTORC1 signaling and substantially protected INS-1E β-cells from LATS2-induced apoptosis (Fig. 7g), further corroborating hyper-activated mTORC1 as downstream player of LATS2 in the context of β-cell apoptosis.

The growth factor (e.g., insulin or IGF), as well as nutrient (amino acid) branches, are two major distinct inputs that regulate mTORC1 activity/localization through small GTPases of the Ras superfamily: Rheb and Rag GTPases. While growth factors control mTORC1 through the PI3K-AKT-TSC-Rheb signaling axis, nutrients such as amino acids act through the Rag family of GTPases^51^. Insulin-dependent AKT activation via PI3K signaling stimulates the GTPase Rheb by blocking the tuberous sclerosis protein complex (TSC), composed of TSC1, TSC2, and TBC1D7 subunits, which function as inhibitor of Rheb. AKT phosphorylates TSC1/2 displacing the TSC complex from the lysosomes, where Rheb is located and functions to activate mTORC1^51, 52^. Instead, amino acid-dependent activation of mTORC1 involves the lysosomal Rag GTPases and recruitment of mTORC1 to the lysosomal membrane to stimulate mTORC1^51^. Of note, glucose-dependent mTORC1 activation also appears to be partially regulated by Rag GTPases, making Rag a “multi-input nutrient sensor”, which signals nutrients to the downstream mTORC1 activation^40, 53^. To examine the effect of LATS2 on key components of the growth factor dependent branch, we overexpressed LATS2 in INS-1E β-cells, which activated mTORC1. LATS2 hyperactivation did not change the level of AKT-Ser473 phosphorylation (important event downstream of PI3K-IRS activation triggered by growth factors) as well as TSC2-Thr1462 phosphorylation (AKT specific site; Supplementary Fig. 4a) suggesting that the AKT-TSC1/2 axis is dispensable for LATS2-induced mTORC1 activation. In contrast, loss- and gain-of-functional experiments using exogenously introduced RagA mutants^54^ showed that LATS2-induced mTORC1 activation is controlled by the amino acid branch. In particular, overexpression of dominant negative GDP bound RagA (HA-RagA-GDP; RagA-T21L) diminished LATS2-induced mTORC1 activation (Supplementary Fig. 4b; HA-metap2 was used as negative control^55^). Conversely, constitutively active GTP bound RagA (HA-RagA-GTP; RagA-Q66L) restored mTORC1 activation in LATS2 depleted INS-1E β-cells treated with high glucose (Supplementary Fig. 4c).

Altogether, these data suggest that LATS2-induced β-cell apoptosis is mediated by Rag-mTORC1 activation.

It has recently been shown that LATS1 phosphorylates Raptor at Ser606, leading to mTORC1 inhibition in HEK293 kidney cells^47^. We took advantage of the phospho-null (S606A) Raptor mutant^47^ to investigate the potential regulation of Ser606-phosphorylation in pancreatic β-cells. As presented in Supplementary Fig. 5a, overexpression of WT-Raptor as well as S606A mutant in INS-1E β-cells similarly activated mTORC1 signaling represented by higher pS6, pS6K, and p4EBP1, compared to control transfected cells. Also, neither WT-Raptor nor S606A mutant altered high glucose induced S6-phosphorylation or apoptosis in INS-1E cells, compared to the high glucose treated control (Supplementary Fig. 5b). All this suggests that Raptor-Ser606 phosphorylation is dispensable for basal mTORC1 activation as well as mTORC1’s deleterious role under glucotoxic conditions in β-cells.

### Bidirectional regulation of LATS2 and autophagy in β-cells

**Fig. 8:**
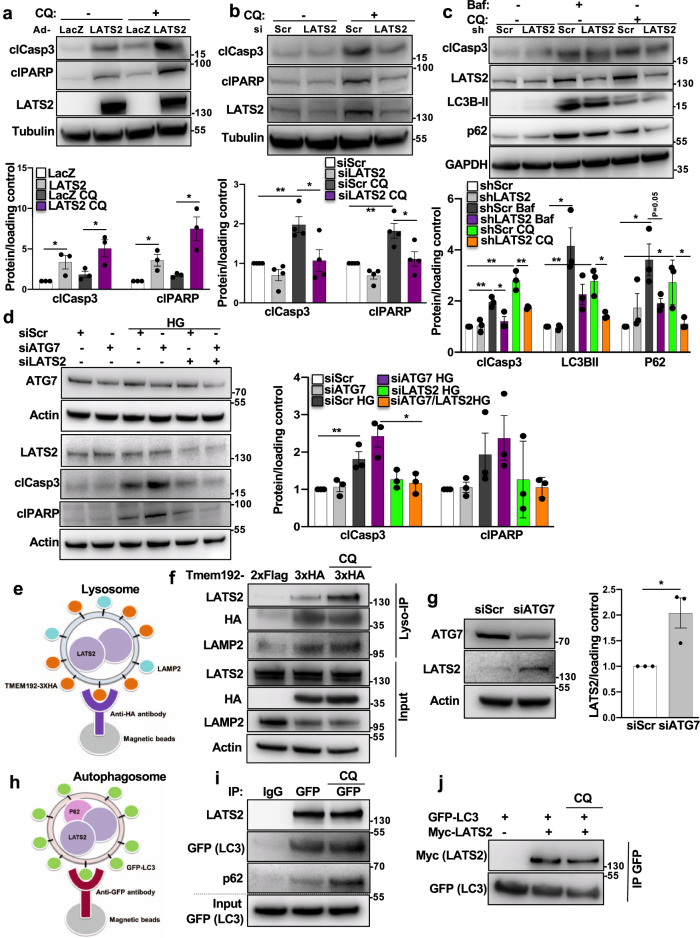
Bidirectional regulation of LATS2 and autophagy in β-cells. **a** Representative western blot and pooled quantitative densitometry analysis (lower panel) of INS-1E cells transduced with Ad-LacZ or Ad-LATS2 and treated with 50 μM Chloroquine (CQ) for 4 h (*n* = 3 independent experiments). **b** Representative western blot and pooled quantitative densitometry analysis (lower panel) of INS-1E cells transfected with siLATS2 or siScr and treated with CQ for 4 h (*n* = 4 independent experiments). **c** Representative western blot and pooled quantitative densitometry analysis (lower panel) of human islets transduced with Ad-hShLATS2 or Ad-shScr and treated with Bafilomycin (Baf) or CQ for 4 h (*n* = 3 different human islets isolations). **d** Representative western blot and pooled quantitative densitometry analysis (right panel) of INS-1E cells transfected with ATG7 siRNA and/or LATS2 siRNA or control siScr and treated with the 22.2 mM glucose for 24 h (*n* = 3 independent experiments). **e** Schematic representation of LysoIP method for immunoprecipitation of intact lysosomes. **f** INS-1E cells were co-transfected with LATS2-Myc and Tmem192-3xHA or Tmem192-2xFlag plasmids for 48 h. One set of cells were treated with 50 µM CQ for last 4 h. Representative western blot of input and lysosomes isolated from INS-1E cells is shown (*n* = 2 independent experiments). **g** Representative western blot and pooled quantitative densitometry analysis (right panel) of INS-1E cells transfected with siScr or siAtg7 for 48 h (*n* = 3 independent experiments). **h** Schematic representation of method for immunoprecipitation of autophagosomes. **i** Stable GFP-LC3 expressing INS-1E cells were transfected with LATS2-Myc plasmid for 48 h. One set of cells were treated with 50 µM CQ for last 4 h. Representative western blot of input and autophagosomes isolated from GFP-LC3 expressing INS-1E cells is shown (*n* = 2 independent experiments). **j** GFP-LC3 expressing INS-1E cells were transfected with or without LATS2-Myc plasmid for 48 h. One set of cells were treated with 50 µM CQ for last 4 h. Representative western blot of immunoprecipitation using anti-GFP magnetic beads is shown (*n* = 2 independent experiments). Data are expressed as means ± SEM. Pooled quantitative densitometry of western blots were normalized to the respective control conditions and ratios, in which a normal distribution of results cannot be proven, were analyzed. *p < 0.05, **p < 0.01; all by two-tailed Student’s *t-*tests.

The nutrient-sensing pathway mTORC1 is the most characterized negative regulator of autophagy and its sustained activation compromises autophagic flux and induces apoptosis in pancreatic β-cells^40, 56^. As LATS2 activates mTORC1 and the latter inhibits a protective-autophagy response in β-cells, we determined the direct impact of blocked autophagy on reduced β-cell viability in the context of LATS2 signaling. We first tested whether gain-/loss-of- LATS2 function could modulate the enhanced cell death under diminished autophagy. INS-1E β-cells and isolated human islets were treated with two different well-established late-stage autophagy inhibitors: BafilomycinA1 (Baf) and Chloroquine (CQ). As expected, suppression of autophagic flux by Baf as well as CQ triggered apoptosis, which was further exacerbated by LATS2 overexpression in INS-1E β-cells (Fig. 8a and Supplementary Fig. 6a). Consistently, also LATS2-overexpressing human islets exhibited increased caspase-3 cleavage (Supplementary Fig. 6b). Conversely, Baf- or CQ-induced β-cell apoptosis was greatly decreased by LATS2 silencing in INS-1E β-cells as well as in human islets (Fig. 8b, c and Supplementary Fig. 6c). Together with exacerbated apoptosis, LATS2 impaired the autophagic flux. Both, the autophagic flux markers microtubule-associated protein 1A/1B-light chain 3 (LC3-II), a key component of the autophagosome membrane, and p62 (also known as SQSTM1), an adapter protein that recruits the cargo proteins into the autophagosome, showed a strong accumulation upon LATS2 overexpression indicating that the forced expression of LATS2 further impaired autophagic flux in human islets (Supplementary Fig. 6b). In contrast, loss of LATS2 attenuated LC3-BII and p62 accumulation induced by autophagy blockers in human islets (Fig. 8c) suggesting a restrictive function of LATS2 in autophagic flux. In line with chemical inhibition of autophagy, knockdown of autophagy-related gene 7 (ATG7), a key component of macroautophagy, exacerbated high glucose-induced apoptosis as represented by increased cleavage of caspase 3 and PARP which is reversed by LATS2 silencing further confirming that LATS2 mediates defective autophagy-induced β-cell apoptosis (Fig. 8d). These results show LATS2 as mediator of defective autophagy-induced apoptosis and autophagic flux in β-cells.

To further assess the effect of LATS2 deficiency on mTORC1 and autophagy, immunohistochemical analyses for pS6 and p62 were performed on pancreatic sections isolated from the HFD-fed mice. HFD-treated control mice (Rip-Cre) showed a markedly higher pS6 in pancreatic islet β-cells compared to the ND mice. This is consistent with the previously reported mTORC1 hyperactivation in HFD diabetic islets by us^41^ and others^57^. In contrast, pS6-expression was normalized in HFD-treated β-LATS2^−/−^ mice (Supplementary Fig. 7a).

In line with our in vitro results in β-cells and human islets, where LATS2 depletion correlated with reduced p62 levels, protein expression of p62 was clearly seen in β-cells in diabetic HFD-fed mice, but not in β-LATS2^−/−^ mice (Supplementary Fig. 7b). These findings show that LATS2 deletion inhibits HFD induced mTORC1 activation as well as p62 accumulation and further support the interplay between LATS2 and the mTORC1-autophagy axis.

We also noticed that endogenous LATS2 protein was increased by autophagy inhibition, suggesting LATS2 as potential substrate for autophagy-mediated degradation (Fig. 8b, c and Supplementary Figs. 6c and 8a). The culminating step for both major autophagy pathways macroautophagy and chaperone-mediated autophagy (CMA) is the degradation of substrate proteins in the lysosomes. Therefore, the presence of a non-lysosomal protein in isolated lysosomes corroborates that the protein is an autophagy substrate. To provide direct evidence for LATS2 not only regulating autophagy, but also vc.vs. for autophagy as regulator of LATS2 protein levels in β-cells, we demonstrated the lysosomal localization of LATS2. Because of the relatively low sensitivity of available specific LATS2 antibodies, immunoprecipitation of endogenous LATS2 was not feasible and therefore, lysosomes of LATS2-overexpressing INS-1E β-cells were isolated by immune-purification, the “Lyso-IP method”^58^. Lysosomes were labeled with the HA-expressing lysosomal membrane protein TMEM192 (TMEM192-3xHA) and anti-HA magnetic beads used for immunoprecipitation of intact lysosomes (Fig. 8e). As a negative control for precipitation, Flag-expressing TMEM192 (TMEM192-2xFLAG) was used. HA-TMEM192-isolated lysosomes represented a highly pure fraction as determined by the absence of markers for other cellular compartments such as mitochondria, ER, Golgi, and endosomes (Supplementary Fig. 8b); 3xHA tagged organelles were successfully isolated seen by the enrichment of HA (TMEM192-3xHA), as compared to the FLAG tagged control (Fig. 8f and Supplementary Fig. 8b). The pulldown efficiency was equal for samples treated with or without CQ as similar levels of HA/TMEM192 as well as LAMP2, the other lysosomal membrane marker, were enriched in both test samples, compared to the Flag precipitated negative control (Fig. 8f). Both the lysosome enriched samples displayed an accumulation of LATS2 protein. LATS2 was considerably higher in the presence of CQ which is unequivocally in line with our previous observation of increased LATS2 by autophagy inhibition. These data suggest LATS2 as substrate for autophagy, as blocking lysosomal degradation by lysosomotropic agents like CQ resulted in the accumulation of LATS2 in the lysosomes. Also, immunofluorescence microscopy of cultured β-cells showed colocalization of LATS2 with LAMP1-containing compartments in CQ-treated cells indicating the lysosomal localization of LATS2 (Supplementary Fig. 8c).

In order to identify the specific type of autophagy pathway that may be involved in the lysosomal degradation of LATS2, we selectively targeted ATG7 and lysosome-associated membrane protein type 2A (LAMP2A) proteins, which are the major essential components of canonical macroautophagy and CMA, respectively. SiRNA mediated genetic downregulation of ATG7 (Fig. 8g) but not of LAMP2A (Supplementary Fig. 8d) induced substantial upregulation of endogenous LATS2 again confirming LATS2 upregulation by autophagy inhibition and supporting macroautophagy as major autophagic mechanism for LATS2 destruction in pancreatic β-cells. In order to corroborate that macroautophagy is involved in the autophagic regulation of LATS2 in β-cells, we isolated autophagosomes from stable GFP-LC3 expressing INS-1E β-cells, which have a GFP tagged to the N-terminus of the autophagosomal membrane protein LC3. The GFP tag on the cytosolic side of the membrane can then be exploited to isolate autophagosomes^59, 60^. Autophagosomes were immunoprecipitated in LATS2-overexpressing INS-1E β-cells using anti-GFP coated magnetic beads (Fig. 8h) in the presence or absence of CQ. LATS2 was enriched in isolated autophagosomes as represented by successful pulldown of GFP (GFP-LC3), compared to IgG control, where LAT2 was absent, indicating the selective LATS2 accumulation in autophagosomes as a direct evidence for LATS2 regulation by macroautophagy (Fig. 8i). While no significant difference was observed in the autophagosomal accumulation of LATS2 by CQ treatment, the level of autophagosomal p62 was higher than without the inhibitor suggesting a cargo-associated accumulation. In addition, the colocalization of LATS2 and LC3 was confirmed by immunofluorescence microscopy in CQ- treated INS-1E cells (Supplementary Fig. 8e).

Another evidence for the macroautophagy-LATS2 interaction comes from direct protein co-immunoprecipitation experiments. Myc-LATS2 was overexpressed in stable GFP-LC3 expressing INS-1E β-cells and anti-GFP magnetic beads were used to immunoprecipitate GFP-LC3 and its potential interacting partners. Subsequent immunoblot analysis revealed that LATS2 co-immunoprecipitated with GFP-LC3 providing an experimental proof for the direct interaction of LC3 and LATS2 (Fig. 8j). We repeated this experiment with normal INS-1E cells, transiently transfected with GFP-LC3 and/or Myc-LATS2 constructs and included all appropriate negative controls (GFP-LC3 alone vs Myc-LATS2 alone). The specific LATS2-LC3 interaction was confirmed and none of the controls showed any unspecific signals (Supplementary Fig. 8f).

It has been shown in multiple cases that the interaction between target proteins (receptors) and LC3-family proteins is mediated by an LC3-interacting region (LIR) motif^61–63^. Therefore, the presence of a LIR would be a potential molecular signature for LC3-interacting proteins. We took advantage of the freely available iLIR database (https://ilir.warwick.ac.uk) developed by Jacomin et al.^64^ and searched for putative canonical LIR motifs in the human LATS2 protein. Based on the in silico analysis of experimentally verified functional LIR motifs, Jacomin et al. redefined the previously described LIR motif- WxxL (where x can be any amino acid) to the 6 amino acids consensus sequence-referred to as the xLIR motif: (ADEFGLPRSK)(DEGMSTV)(WFY) (DEILQTV)(ADEFHIKLMPSTV)(ILV), where the residues marked in bold (positions 3 and 6) correspond to the important residues for the interaction with LC3-family proteins^64^. Our in silico analysis identified one complex xLIR motif as well as more than ten LIR motifs- WxxL (Supplementary Fig. 9a, b) supporting our co-IP data which showed interaction between LATS2 and LC3. Further experimental investigation using LIR-inactive LATS2 mutants is required to verify this. Altogether, our data suggest the existence of a mutual regulatory axis between LATS2 and autophagy to fine-tune the β-cell apoptosis program.

## Discussion

The pancreatic β-cell’s vast metabolic plasticity as well as its stress response to cope with high metabolic demands and subsequent pro-diabetic signals is directed by the structure and spatiotemporal dynamics of complex signal transduction networks. Diabetes-associated perturbations in these orchestrated networks occur at various levels, resulting in the dysregulation of physiological functional β-cell mass adaptation. Our work provides direct evidence that Hippo pathway’s central kinase LATS2 is activated under diabetogenic conditions, which induced β-cell failure through increased β-cell apoptosis and impaired β-cell function, while LATS2’s inactivation resulted in resistance to β-cell apoptosis, improved glycemia, insulin secretion and β-cell mass in in vitro, ex vivo and in vivo experimental models of diabetes.

We identified LATS2 as a key upstream activator of mTORC1 in stressed β-cells in which mTORC1 inhibition blocked β-cell apoptosis, suggesting that the pro-apoptotic action of LATS2 is mTORC1-dependent. While LATS2 activated mTORC1 in β-cells, LATS kinases can also do the contrary in a different cellular context, namely suppress mTORC1 by phosphorylating Raptor at Serine 606 and subsequently impairing the Raptor downstream interaction with Rheb^47^. There are several possible explanations for such distinct effect: (i) LATS1 and LATS2 kinases do not necessarily share redundant functions in all cell types. In the Gan et al. study, the majority of biochemical and functional experiments have been performed in the model HEK293 kidney cell line, and not in primary cells, or other cell types and thus, a unifying nature for the proposed mechanism was not provided. This is supported by data from the homozygous phosphomimetic (S606D; *Raptor*^D/D^) knock-in mice, in which the LATS mediated Raptor-S606 phosphorylation site is constitutively active. Diminished mTORC1 signaling shown in the liver, heart and kidneys but not in the spleen and brain indeed suggests a tissue or cell type dependent regulation of mTORC1 by LATS kinases. (ii) Another explanation may arise from a different cellular context in our and Gan’s et al. studies. While contact inhibition -as a result of high cell density- is a driving force and critical factor for the LATS1/2 mediated mTORC1 suppression, LATS2 induced mTORC1 hyperactivation under diabetogenic conditions of stress and nutrient overload in β-cells is likely to be independent of cell density. (iii) Additionally, LATS mediated phosphorylation of Raptor reduces growth factor induced mTORC1 stimulation through Rheb^47^, while we show here that LATS2-induced mTORC1 activation is regulated, at least in part, by the other amino acid branch of mTORC1 activation through its key component Rag-GTPase.

**Fig. 9:**
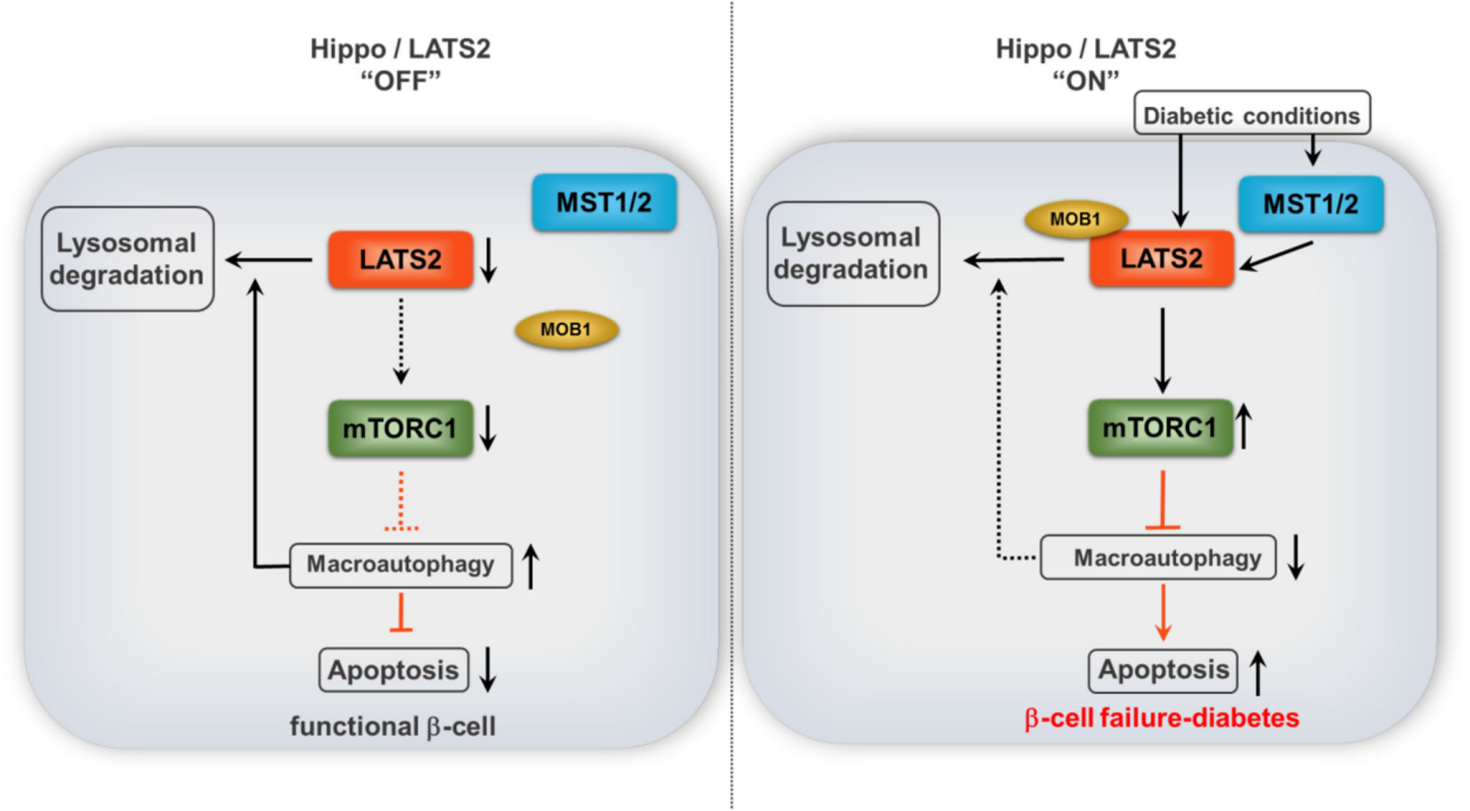
Proposed model of LATS2 action in β-cells. LATS2, mTORC1, and autophagy may constitute a stress-sensitive survival pathway. Under physiological (Hippo”OFF”) or acute stress conditions, autophagy promotes β-cell survival by directly degrading LATS2 and consequently reinforcing protective-autophagy mechanism through a positive-feedback loop. However, prolonged diabetogenic stress activated LATS2 (Hippo “ON”) leading to mTORC1 hyper-activation, defective autophagy, ultimately further LATS2 accumulation and subsequent β-cell apoptosis. LATS2 (Large tumor suppressor 2; in orange), MST1/2 (Mammalian Sterile 20-like kinases 1/2; in blue), MOB1 (Mps-one binder 1; in beige), mTORC1 (Mammalian target of rapamycin complex 1; in green).

Downstream of mTORC1, autophagy is a key process for maintaining β-cell viability and functional homeostasis^40^. Defective autophagy is a hallmark of β-cell failure in T2D^65–68^. Our current study expands a previously unrecognized mechanistic link between LATS, mTORC1, and autophagy. Firstly, LATS2 controls the autophagic flux and autophagy-induced apoptosis through the regulation of mTORC1. Secondly, autophagy itself regulates LATS2’s protein turnover by directly targeting LATS2. This suggests that LATS2, mTORC1 and autophagy may constitute a stress-sensitive survival pathway (Fig. 9). Under acute stress conditions, autophagy promotes β-cell survival by directly degrading LATS2 and consequently reinforcing protective-autophagy mechanism through a positive-feedback loop. However, prolonged stress activated LATS2 leading to mTORC1 hyper-activation, defective autophagy, ultimately further LATS2 accumulation and subsequent β-cell apoptosis. This antagonism between LATS2 and autophagy suggests that the outcome of the mutual regulation of both pathways under conditions of increased β-cell stress and demand in a diabetic microenvironment is probably determined by the extent and duration of activated LATS2. In this context, aberrant LATS2 activity triggered by diabetic stimuli may shift the balance towards chronic mTORC1 activation, resulting in defective autophagic flux and β-cell apoptosis. This highlights the existence of a functional bidirectional cross-communication between LATS2 and autophagy for the regulation of β-cell viability under physiological conditions and uncovers LATS2 to mediate a cross-talk between the Hippo pathway and autophagy.

The Hippo pathway and autophagy share a complex network with bidirectional connections tightly controlling the autophagic flux in response to extracellular cues, nutrients availability, and metabolic adaptations to direct cellular and systemic homeostasis^69^. For instance, LATS1/2 upstream kinases MST1/2 were shown to differentially regulate autophagic outcomes. While MST1 directly phosphorylates LC3 to foster autophagosomes-lysosomes fusion and autophagic flux for intracellular cargo clearance (such as bacteria) in mouse-embryonic fibroblasts (MEFs)^70^, it phosphorylates the autophagy regulator Beclin1, subsequently inhibiting the Beclin1-Vps34 complex, which leads to autophagy inhibition in cardiomyocytes^71^ suggesting contextual and perhaps cell-type dependent regulation of autophagy.

Downstream target of LATS1/2 kinases is the Hippo effector, the transcriptional co-activator YAP, which itself is also strongly linked to autophagy^69^. Like LATS2 (but with opposite functional outcomes), YAP not only functions as upstream regulator of autophagy by regulating the degradation of autophagosomes as well as the maturation of autophagosomes^72, 73^, but also acts as an autophagic substrate in a way that the autophagic flux triggers protein turnover of YAP and its targets^74, 75^. During embryonic development, YAP is highly expressed throughout the pancreas in a multipotent progenitor stage^13, 16–18, 33^. However, after differentiation, YAP remains expressed in ductal and acinar cells^18^, but is excluded from β-cells and other endocrine islet cells at the time when the key endocrine progenitor transcription factor and marker Ngn3 emerges^19^ coinciding with their very low proliferative capacity. Re-expression of the “disallowed“ YAP in human islets specifically fosters β-cell proliferation^19, 76^. Single cell RNA-seq data (for example, ref. ^77^), as well as gene and protein functional expression analyses^17–19, 76^, confirm that YAP is not expressed in terminally differentiated mature primary human and mouse islets and β-cell lines. While YAP signals are excluded as Hippo targets in mature islets and β-cells, functional MST1/2 and LATS1/2 kinases operate Hippo signaling in the absence of YAP directing alternative Hippo downstream effector(s). Thus, in our experimental setting, LATS2 maneuvers its downstream events, i.e., apoptosis, impaired insulin secretion by a crosstalk with autophagy signals through non-canonical YAP independent mechanisms in β-cells. Such YAP-independent actions of LATS have also been reported in other cellular contexts; e.g., the mitotic spindle orientation in *Drosophila*^78^ or the regulation of mTORC1 signaling in mammals^47^ in which YAP knockdown has insignificant effects on mTORC1 activity, indicating that the Hippo/LATS pathway primarily regulates mTORC1 independent of YAP. While we could show by multiple experimental settings that LATS-induced effects on β-cell apoptosis and autophagy is mediated by mTORC1 activation, it is equally possible that LATS2 activation and mTORC1 hyperactivation act in parallel under stress- and diabetogenic conditions to dysregulate the β-cell compensatory machinery and induce β-cell death, dysfunction and thus metabolic failure and diabetes.

The role of the Hippo pathway in human disease, especially in cancer has been substantiated by a robust line of research over the past 10 years^9, 10, 79^. Using a multi-model approach, we have identified LATS2 as pro-apoptotic kinase whose abnormal activation led to impaired β-cell survival and function, while its depletion restored functional β-cell mass and protected against diabetes progression. Blocking LATS2 could be a promising strategy to improve β-cell survival in diabetes.

## Methods

### Cell culture, treatment, and islet isolation

Human islets were isolated from pancreases of non-diabetic organ donors at the Universities of Illinois at Chicago, Wisconsin, Lille or ProdoLabs and cultured on extra cellular matrix (ECM)-coated dishes (Novamed, Jerusalem, Israel)^80^ or on Biocoat Collagen I coated dishes (#356400, Corning, ME, USA). Human islets were cultured in complete CMRL-1066 (Invitrogen) medium at 5.5 mM glucose and mouse islets, the clonal rat β-cell line INS-1E, and the human insulinoma CM cell line in complete RPMI-1640 medium at 11.1 mM glucose^14, 81^. Islets from β-cell specific LATS2 knockout (β-LATS2^−/−^) and control mice were isolated by pancreas perfusion with a Liberase TM (#05401119001, Roche, Mannheim, Germany) solution^80^ according to the manufacturer’s instructions and digested at 37 °C, followed by washing and handpicking. Human and mouse islets and INS-1E cells were exposed to complex diabetogenic conditions: 22.2 mM glucose, 0.5 mM palmitic acid^82^, the mixture of 2 ng/mL recombinant human IL-1β (R&D Systems, Minneapolis, MN) plus 1,000 U/ml recombinant human IFN-γ (PeProTech) for 24-72 h. In some experiments, cells and islets were additionally cultured with 100 nM Rapamycin or 10 μM S6K1 selective inhibitor PF-4708671 (Calbiochem) for 24 h, 50 μM chloroquine (Sigma), 20 nM Bafilomycin (Sigma) or a cocktail of Leupeptin (Sigma) and NH_4_Cl for 12 h.

Human islets were distributed by the two JDRF and NIH supported approved coordination programs in Europe (Islet for Basic Research program; European Consortium for Islet Transplantation ECIT) in agreement with the French Regulations and the Institutional Ethical Committee (“Comité d’Ethique du Centre Hospitialier Régional et Universitaire de Lille”) and in the US (Integrated Islet Distribution Program IIDP)^83^, which is, in collaboration with the islet isolation centers, responsible for obtaining consent from donors. The IIDP requires informed consent for research use by the donor or the donor’s legal representative. Every islet isolation offered through the IIDP is designated by the isolation centers as having received sufficient documentation of consent of research use of the pancreas.

Organ donors are not identifiable and anonymous. This work includes islet cells from adult brain-deceased donors insufficient in number for clinical transplantation with informed consent (USA) or when there has been no written refusal for organ transplantation after verification there has been no written refusal (national refusal registry) for research; verbal consent for research donation given is requested from next of kin (Europe; France). France has presumed consent legislation in place for deceased donors. All human islet experiments were performed in the islet biology laboratory, University of Bremen. Ethical approval for the use of human islets in this project had been granted by the Ethics Committee of the University of Bremen. The study complied with all relevant ethical regulations for work with human cells for research purposes.

### Mice

β-cell-specific LATS2 knockout (β-LATS2^−/−^) mice were generated by crossing mice harboring exon 4 of the LATS2 gene flanked by *loxP* sites (LATS2^fl/fl^, provided by Dr. Dae-Sik Lim, Korea Advanced Institute of Science and Technology, South Korea^84^) with C57BL/6J strain mice expressing Cre under the rat insulin-2 promoter (B6;D2-Tg(Ins-cre)23Herr:RIP-Cre^85^, kindly provided by Susanne Ullrich (Medizinische Klinik, Universitätsklinikum, Tübingen). RIP-Cre-LATS2^fl/+^ mice on the C57BL/6J background were intercrossed to generate RIP-Cre-LATS2^fl/fl^ (β-LATS2^−/−^). For multiple low dose streptozotocin (MLD-STZ) experiments, 8- to 10-week old β-LATS2^−/−^, flox control (LATS2^fl/fl^) and flox-negative littermates (RIP-Cre) were injected with STZ for 5 consecutive days (40 mg/kg STZ or citrate buffer vehicle control). For the high fat diet (HFD) experiments, 8-week old β-LATS2^−/−^ mice and Rip-Cre controls were fed a normal diet (ND, Harlan Teklad Rodent Diet 8604, containing 12.2, 57.6, and 30.2% calories from fat, carbohydrate, and protein, respectively) or a high fat/ high sucrose diet (HFD, “Surwit” Research Diets, New Brunswick, NJ, containing 58, 26, and 16% calories from fat, carbohydrate and protein, respectively^86^) for 17 weeks. For both models, random blood was obtained from the tail vein of non-fasted mice and glucose was measured using a Glucometer (Freestyle; TheraSense Inc., Alameda, CA). Heterozygous leptin receptor-deficient mice on the C57BLKS/J background (Lepr^db/+^, db/^+^) were purchased from Jackson Laboratory. By breeding of these mice, we obtained diabetic Lepr^db/db^ (db/db) as well as non-diabetic heterozygous Lepr^db/+^ (db/+) mice. For data presented in Fig. 1 and Supplementary Fig. 1, islets were isolated after 16 weeks of HFD or at the age of 12 weeks (db/db). Mice were killed and pancreases isolated at the end of experiment. All mice used in this experiment were male and housed in a temperature-controlled room with a 12-h light–dark cycle and were allowed free access to food and water in agreement with NIH animal care guidelines, §8 German animal protection law, German animal welfare legislation and with the guidelines of the Society of Laboratory Animals (GV-SOLAS) and the Federation of Laboratory Animal Science Associations (FELASA). All protocols were approved by the Bremen Senate (Senator for Science, Health and consumer protection) and we have complied with all relevant ethical regulations for animal testing and research.

### Glucose and insulin tolerance tests and measurement of insulin release

For ipGTTs of control and hyperglycemic HFD/STZ-treated mice, mice were fasted 12 h overnight and injected with glucose (40%; B. Braun, Melsungen, Germany) at a dose of 1 g/kg body weight according to an established protocol^87^. Blood samples were obtained at time points 0, 15, 30, 60, 90, and 120 min for glucose measurements using a glucometer. For i.p. insulin tolerance tests, mice were injected with 0.75 U/kg body weight recombinant human insulin (Novolin, Novo Nordisk) after 4–5-h fasting, and glucose concentration was determined with the Glucometer. Insulin secretion was measured before (0 min) and after (15 and 30 min) i.p. injection of glucose (2 g/kg body weight) and measured using ultrasensitive mouse Elisa kit (ALPCO Diagnostics, Salem, NH).

### Plasmids and siRNAs

Myc-LATS2 and kinase dead Myc-LATS2 (LATS2-KD) were provided by Dr. Jixin Dong (Nebraska Medical Center, Omaha, NE)^37^. Raptor-WT and Raptor-S606A mutant were kindly provided by Wenjian Gan (Medical University of South Carolina, SC, USA) and Wenyi Wei (Harvard Medical School, Boston, MA, USA)^47^. pRK5-HA GST RagA 66L was a gift from David Sabatini (Addgene plasmid # 19300; RRID:Addgene_19300)^54^. pRK5-HA GST RagA 21L was a gift from David Sabatini (Addgene plasmid # 19299; RRID:Addgene_19299)^54^. pBABEpuro GFP-LC3 was a gift from Jayanta Debnath (Addgene plasmid # 22405; http://n2t.net/addgene:22405; RRID:Addgene_22405)^88^. pCDNA3.1neo-NLucYAP15 and pCDNA3.1neo-14-3-3-CLuc (LATS-BS) were gift from Xiaolong Yang (Addgene plasmid # 107610; RRID:Addgene_107610)^34^. pLJC5-Tmem192-3xHA and pLJC5-Tmem192-2xFlag were gifts from David Sabatini (Addgene plasmid # 102930; RRID:Addgene_102930). pRK5-HA-metap2 was a gift from David Sabatini (Addgene plasmid # 100512; RRID:Addgene_100512)^89^. GFP or metap2 was used as a control. All siRNAs were purchased from Dharmacon. A mix of ON-TARGETplus siRNAs directed against human LATS2 (26524) sequences GAAGUGAACCGGAAAUGC, AAUCAGAUAUUCCUUGUUG, ACACUCACCUCGCCCAAUA, GCACGCAUUUUACGAAUUC, rat LATS2 (305922) sequences GGAAAUAGCCGGCAGCGAC, UCAAUAAUGACUUGUACGA, GCAGGUUCUUCGACGACAA, ACCAGAAGGAGUCGAACUA, rat LATS1 (308265) sequences CCGAAAACCUGGCACGAUU, AUCCAAAGCCCAUCGAAUA, CAAGAAAAGUCGAUACGAA, GAGCGAUGGUAACGAGGAA, rat MOB1a (297387) sequences GGAAUGACGGUUAGGUAA, CAUACUAAAUAUAGCGUCU, AGUCAGUACUUGAUUAU, CCGAUUGACUGGUGAAUUC, rat Raptor (287871) sequences GAGCUUGACUCCAGUUCGA, GCUAGGAACCUGAACAAAU, GCACACAGCAUGGGUGGUA, GAAUCAUGAGGUGGUAUAA, and rat Atg7 (312647), sequences CAAAGUUAACAGUCGGUGU, AGUGAAUGCCAGCGGGUUC, CUGGAGGAACUCAUCGAUA, CCCAGAAGAAGUUGAACGA. Four different custom siRNAs were designed against Lamp2A, sequences siLAMP2A#1- GCGCCAUCAUACUGGAUAUUU, siLAMP2A#2- GUGCAGAUGAAGACAACUUUU, siLAMP2A#3- GGGAGGAGUACUUAUUCUAUU, and siLAMP2A#4- AGAGUAUUCUACAGCUCAAUU. A second mix of siRNAs directed against rat LATS2 (siGENOME; 305922) sequences GGAACAGCCUCAAUAAUGA, GGAAACAGCCUGCACCCUA, GAAGUUUGGACCUUAUCAA, AAGUGUGCCUUGCCUGUUAA. An ON-TARGETplus non-targeting siRNA pool from Dharmacon served as a control.

### Transfections

LATS2, LATS2-KD, LATS-BS, LC3, pLJC5-Tmem192-3xHA, pLJC5-Tmem192-2xFlag, Raptor-WT, Raptor-S606A, HA-RagA-T21L, HA-RagA-Q66L, HA-metap2, and GFP plasmids were used to overexpress these proteins in INS-1E cell. 100 nM siRNAs were used for the transfection in human islets and INS-1E cells^14^. To deliver desired siRNA/DNA into dispersed isolated islets as well as INS-1E cells two different transfection methods were used. (1) In brief, isolated islets or INS-1E cells were pre-incubated in transfection Ca^2+^-KRH medium for 1 h; and then lipoplexes (Lipofectamine 2000, Invitrogen)/siRNA ratio 1:20 pmol or lipoplexes/DNA ratio 2.5:1) were added to islets or INS-1E cells; after an additional 4–6 h incubation, CMRL-1066 or RPMI-1640 medium containing 20% FCS was added to the transfected islets or INS-1E cells. (2) jetPRIME^®^ transfection reagent (#114-75; Polyplus transfection, France) was mixed with jetPRIME buffer and siRNA/DNA according to the manufacturer’s instructions. The jetPRIME-siRNA/DNA complexes were then added to complete CMRL-1066 or RPMI-1640 to transfect dispersed human islets or INS-1E cells. Efficient transfection was evaluated based on western blot, qPCR, and fluorescent microscopy.

### Adenovirus transduction

The adenoviruses Ad-h-LATS2 expressing human LATS2 and Ad-GFP-U6-hLATS2-shRNA expressing GFP and human LATS2 shRNA were obtained from Vector Biolabs. The sequence for the shRNA of LATS2 was:

CCGG-CTACTCGCCATACGCCTTTAACTCGAGTTAAAGGCGTATGGCGAGTAG-TTTTTG. Ad-LacZ or Ad-GFP-U6-shRNA were used as respective controls. For transduction, human islets or INS-1E cells were plated for 24 h; then infected at a multiplicity of infection (MOI) of 20 (for INS-1E) or 100 (for human islets) for 4 h in CMRL/RPMI medium without FCS. After 4 h incubation, human islets or INS-1E cells were washed with medium and incubated for an additional 48 h or treated with 22.2 mM glucose alone/ plus palmitate, or 2 ng/mL IL1-β plus 1000 U/mL IFN-γ.

### Glucose-stimulated insulin secretion (GSIS)

Glucose-stimulated insulin secretion was performed by pre-incubating primary human islets in Krebs-Ringer bicarbonate buffer (KRB) containing 2.8 mM glucose for 30 min, followed by KRB buffer containing 2.8 mM glucose for 1 h (basal) and then an additional 1 h in KRB containing 16.7 mM glucose (stimulated). Islets were washed with PBS and lysed with RIPA buffer to extract protein. Insulin was determined using human insulin ELISA (ALPCO Diagnostics, Salem, NH). Secreted insulin was normalized to insulin content.

### Immunohistochemistry

Mouse pancreases were dissected and fixed in 4% formaldehyde at 4 °C for 12 h before embedding in paraffin^80^. 4-µm sections were deparaffinized, rehydrated, and incubated overnight at 4 °C with anti-Ki67 (Dako; #M7249) or guinea pig anti-P62 (GP62-C) from PROGEN or anti-pS6 (CST; 4858) in combination with TSA (Invitrogen #T30955) and for 2 h at room temperature with anti-insulin (Dako; A0546) antibodies (all at a dilution of 1:100) followed by Cy3-conjugated donkey anti-rat (712-165-150), or anti-guinea pig (706-165-148), or anti-rabbit (711-165-152) and fluorescein isothiocyanate (FITC)- conjugated donkey anti-guinea pig (706-096-148) (all from Jackson ImmunoResearch Laboratories, West Grove, PA; 1:200 dilution) for 1 h at RT. Slides were mounted with Vectashield with 4′6-diamidino-2-phenylindole (DAPI) (Vector Labs). β-cell apoptosis in Bouin’s fixed isolated human islets or mouse pancreatic sections was analyzed by the terminal deoxynucleotidyl transferase-mediated dUTP nick-end labeling (TUNEL) technique according to the manufacturer’s instructions (In Situ Cell Death Detection Kit, TMR red; Roche) and double stained for insulin. Fluorescence was analyzed using a Nikon MEA53200 (Nikon GmbH, Dusseldorf, Germany) microscope and images were acquired using NIS-Elements software (Nikon).

For autophagy analyses, INS-1E β-cells and the human β-cell line CM were seeded on gelatin coated glass coverslips in 24-well plates and then transfected with GFP-LC3 and Myc-LATS2 plasmids or with the Myc-LATS2 plasmid alone using jetPRIME^®^ transfection reagent. After 24 h, cells were treated with 100 µM Chloroquine for 6 h to inhibit autophagosome degradation. Slides were rinsed with PBS once and fixed for 30 min with 4% paraformaldehyde in PBS at RT followed by 4 min permeabilization with 0.5 % TritonX-100 in PBS. After rinsing twice with PBS, cells were blocked with blocking buffer containing 3% BSA and then incubated overnight at 4 °C with GFP (CST; #2956, 1:100) and Myc (CST; #2276, 1:700) primary antibodies followed by the secondary antibodies: Cy3-conjugated donkey anti-mouse (715-165-150) or fluorescein isothiocyanate (FITC)-conjugated donkey anti-rabbit (711-096-152); (all from Jackson ImmunoResearch Laboratories, West Grove, PA; 1:200 dilution) for 1 h at RT. Co-staining of LATS2 and lysosomal marker LAMP1 was performed using Myc (CST; #2276, 1:700) and Alexa Fluor^®^488**-**conjugated LAMP1 (CST; #58996, 1:50) antibodies and anti-mouse Cy3-conjugated secondary antibody (for Myc). Coverslips were mounted on glass slides using Vectashield with 4’6-diamidino-2-phenylindole (DAPI; Vector Labs). Confocal analyses were performed with an LSM880 ZEISS confocal laser scanning microscope (Zeiss, Jena, Germany).

### Morphometric analysis

For morphometric data, ten sections (spanning the width of the pancreas) per mouse were analyzed. Pancreatic tissue area and insulin-positive area were determined by computer-assisted measurements using a Nikon MEA53200 (Nikon GmbH, Dusseldorf, Germany) microscope and images were acquired using NIS-Elements software (Nikon). β-cell mass was obtained by multiplying the β-cell fraction by the weight of the pancreas^14^.

### Western blot analysis

Human or mouse islets and INS-1E cells were washed twice with ice-cold PBS and lysed with RIPA lysis buffer containing Protease and Phosphatase Inhibitors (Pierce, Rockford, IL, USA). Protein concentrations were measured by the BCA protein assay (Pierce). Lysates were fractionated by NuPAGE 4–12% Bis-Tris gel (Invitrogen) and electrically transferred into PVDF membranes. Membranes were blocked in 2.5% non-fat dry milk (CST) and 2.5% BSA (Sigma) for 1 h at room temperature and incubated overnight at 4 °C with the following antibodies: rabbit anti-LATS2 (5888), rabbit anti-LATS1 (9153), mouse anti-Myc (2276), rabbit anti-cleaved caspase-3 (9664), rabbit anti-PARP (9542), rabbit anti-Cleaved PARP (9545), rabbit anti-Raptor (2280), rabbit anti-phosho-p70 S6 Kinase (9234), rabbit anti- phosho-S6 ribosomal protein (4858), rabbit anti-phosho-4EBP1 (2855), rabbit anti-LC3B (2775), rabbit anti-Atg7 (8558), rabbit anti-GFP (2956), rabbit anti-HA (2367), rabbit anti-phospho-Tuberin/TSC2 (3611), rabbit anti-Tuberin/TSC2 (4308), rabbit anti-pAKT (4058), rabbit anti-AKT (9272), mouse anti-S6 ribosomal protein (2317), rabbit anti-p70 S6 Kinase (2708), rabbit anti-4EBP1 (9644), rabbit anti-tubulin (2146), rabbit anti-glyceraldehyde 3-phosphate dehydrogenase (2118) and rabbit anti-β-actin (4967) all from Cell signaling technology (CST) and guinea pig anti-P62 (GP62-C) from PROGEN and rabbit anti-LAMP2A (AB10971511) from Abcam. Organelle identification markers data presented in Supplementary Fig. 8b were detected using antibodies from the Organelle Localization IF Antibody Sampler Kit (CST; 8653). Primary antibodies were followed by horseradish-peroxidase-linked anti-rabbit (111-035-003), anti-mouse (115-035-003) or anti-guinea pig (106-035-003) secondary antibodies (all from Jackson; 1:3000 dilution). All primary antibodies were used at 1:1000 dilution in Tris-buffered saline plus Tween-20 (TBS-T) containing 5% BSA. Membrane was developed using a chemiluminescence assay system (Pierce) and analyzed using DocITLS image acquisition 6.6a (UVP BioImaging Systems, Upland, CA, USA). Uncropped and unprocessed scans of all blots are provided in the Source data file.

### LATS-BS luciferase assay

INS1-E cells or isolated mouse islets were transfected with LATS-BS firefly luciferase reporter constructs using jetPRIME transfection reagent (PolyPlus, Illkirch, France). As internal transfection control, pRL-Renilla luciferase control reporter vector (Promega) was co-transfected into each sample. 24 h after transfection, INS-1E cells were treated with 22.2 mM glucose alone or plus palmitate for another 24 h. Thereafter, Western blot analysis (see above) and luciferase assay was performed using Dual-Luciferase Reporter Assay System (Promega)^89^ in a parallel set of experiments. Luciferase signal was calculated based on the ratio of luciferase activity of LATS-BS to control reporter vector.

### Lyso-IP

Approx. 30 million INS-1E cells were used for each condition. Each dish was transfected with Tmem192-2XFlag/Tmem192-3XHA and LATS2-Myc plasmids after an adapted previously well-established protocol for the isolation of lysosomes^58^. INS-1E cells were washed with ice-cold PBS and collected in 1 ml KPBS (136 mM KCl, 10 mM KH_2_PO_4_, [pH 7.25 adjusted with KOH]) supplemented with Protease and Phosphatase Inhibitors and centrifuged at 1000 × *g* for 2 min at 4 °C. Pellet was resuspended in 950 μl of KPBS and 25 μl suspension was saved for the input. The remaining cell suspension was homogenized with 60 strokes of a dounce homogenizer (125 rpm, setting 1). Lysate was centrifuged at 1000 × *g* for 2 min at 4 °C. The supernatant was incubated with 25 µl of KPBS prewashed anti-HA magnetic beads (ThermoFischer) on rotation at 4 °C for 15 min. Beads were separated using a magnet and immuno-captured lysosomes were gently washed 4 times with 1 mL KPBS on rotation at 4 °C for 4 min each. Proteins were extracted from bound lysosomes directly by adding 2× loading buffer followed by heating at 95 °C for 10 min. Beads were separated, sample was spun shortly and collected for immunoblot analysis, for which half of the eluted sample was loaded on the gel (approximately 50–60× enrichment of the loaded input).

### Isolation of autophagosomes

An adapted protocol from ref. ^59^ has been used for the isolation of autophagosomes. Approximately 20 million stable GFP-LC3 expressing INS-1E β-cells were used per condition. Cells were washed twice with PBS, scrapped in 2 ml ice-cold PBS and centrifuged at 1000 × *g* for 3 min at 4 °C. Pellet was resuspend in 1 ml cold PBS and 50 µl was removed for input. The remaining suspension was centrifuged at 1000 × *g* for 3 min at 4 °C and pellet was resuspend in 1 ml ice-cold resuspension buffer (0.25 M sucrose, 1 mM EDTA, and 10 mM HEPES-NaOH [pH 7.4]). Cell were lysed by a Dounce homogenizer (60 strokes, 125 rpm, 1st setting) followed by centrifugation at 1000 × *g* for 10 min at 4 °C to remove cell debris and nucleus. The supernatant was then centrifuged at 20,000 × *g* for 20 min at 4 °C to enrich autophagosomes. Pellet containing autophagosomes were resuspended in resuspension buffer and incubated with equilibrated anti-GFP-Trap^®^ Magnetic beads (Chromotek, Planegg, Germany) or rabbit IgG (CST) conjugated magnetic beads (25 µl) for 2 h at 4 °C on rotation. The beads were separated using a magnet and washed four times 4 min each with wash buffer (resuspension buffer supplemented with 0.15 M NaCl) at 4 °C. The beads were separated and boiled in 2× SDS sample buffer at 95 °C for 10 min and spun down. Beads were separated and the sample was centrifuged at high speed for 5 min and collected for immunoblot analysis.

### Co-immunoprecipitation

Protocol adapted from GFP-Trap Magnetic Agarose Kit (Chromotek, Planegg, Germany). For one immunoprecipitation reaction, approximately 10 million GFP-LC3 expressing INS-1E cells or normal INS-1E cells were used. INS-1E cells were washed twice with PBS, scrapped in 2 ml ice-cold PBS and centrifuged at 1000 × *g* for 3 min at 4 °C. Pellet was resuspended in 200 µl ice-cold lysis buffer (10 mM Tris/Cl pH 7.5, 150 mM NaCl, 0.5 mM EDTA, 0.5 % Nonidet™ P40 Substitute, 0.09% sodium azide) supplemented with Protease and Phosphatase Inhibitors. The tube was placed on ice for 30 min with extensively pipetting every 10 min. Cell lysate was centrifuged at 15,000 × *g* for 15 min at 4 °C and 300 μl dilution buffer (10 mM Tris/Cl pH 7.5, 150 mM NaCl, 0.5 mM EDTA, 0.018% sodium azide) supplemented with Protease and Phosphatase Inhibitors was added to the supernatant. 50 µl of the suspension was saved for input. The remaining suspension was incubated with equilibrated anti-GFP conjugated magnetic beads (25 µl) for 1 h at 4 °C on rotation. The beads were separated using a magnet and washed four times 4 min each with dilution buffer at 4 °C. The beads were separated and boiled in 2× SDS sample buffer at 95 °C for 10 min and spun down. Beads were separated and the sample was collected for immunoblot analysis.

### Generation of INS-1E cell with stable GFP-LC3 expression

To overexpress GFP-LC3 in INS-1E cells, cultured cells were transfected with pBABEpuro GFP-LC3 and selected with 1.5–3 μg/mL puromycin. Resistant colonies were identified by GFP under fluorescent microscopy and used for further experiments. After selection, INS-1E cells were maintained in culture medium containing 2 μg/ml puromycin.

### Genomic PCR

Genomic DNA was extracted from liver, heart, spleen, kidney, hypothalamus, and isolated pancreatic islets according to the manufacturer’s instruction (DNeasy^®^ Blood&Tissue Kit, QIAGEN). Genotyping was performed using the following primers: flox-F 5′ CCG GAG TCA TTG CTT GTT TT 3′, flox-R 5′ GGA GAT CCT GGG TAC TGC AC 3′, flox-F del 5′ ACA TGA CAC TAC GGG GCC TAG C 3′^84^. A 300 bp band was amplified from floxed mice and 400 bp band was amplified if the LATS2 gene was deleted.

### Statistical analyses

To perform statistical analysis, at least 3 independent experiments were performed for the human islets (3 different donors) and INS-1E cells, as reported in all figure legends. Data are presented as means ± SEM. Mean differences were determined by two-tailed Student’s *t*-tests or one-way ANOVA for multiple group comparisons with Tukey’s post hoc test. Densitometry analyses of western blot bands were examined by two-tailed Student’s *t*-tests. Results were normalized to the respective control conditions and ratios analyzed, in which a normal distribution of results cannot be proven. *P* value < 0.05 was considered statistically significant.

### Reporting summary

Further information on research design is available in the Nature Research Reporting Summary linked to this article.

## Supplementary information

Supplementary Information File

Reporting Summary

## Data Availability

All data generated or analyzed during this study are included in this article and its Supplementary information files. All original source data are provided as a Source Data file. Source data are provided with this paper.

## Source data

Source data
