# Supplementary material for: The Hippo kinase LATS2 impairs pancreatic β-cell survival in diabetes through the mTORC1-autophagy axis
Source: Nat Commun. 2021 Aug 13;12:4928. doi: 10.1038/s41467-021-25145-x (PMC8363615; doi:10.1038/s41467-021-25145-x)
Supplement: Supplementary file 1 — Supplementary Information File [file 41467_2021_25145_MOESM1_ESM.pdf]

Supplementary Information

The Hippo kinase LATS2 impairs pancreatic β-cell survival in diabetes through the mTORC1-autophagy axis

Ting Yuan, Karthika Annamalai, *et al.*

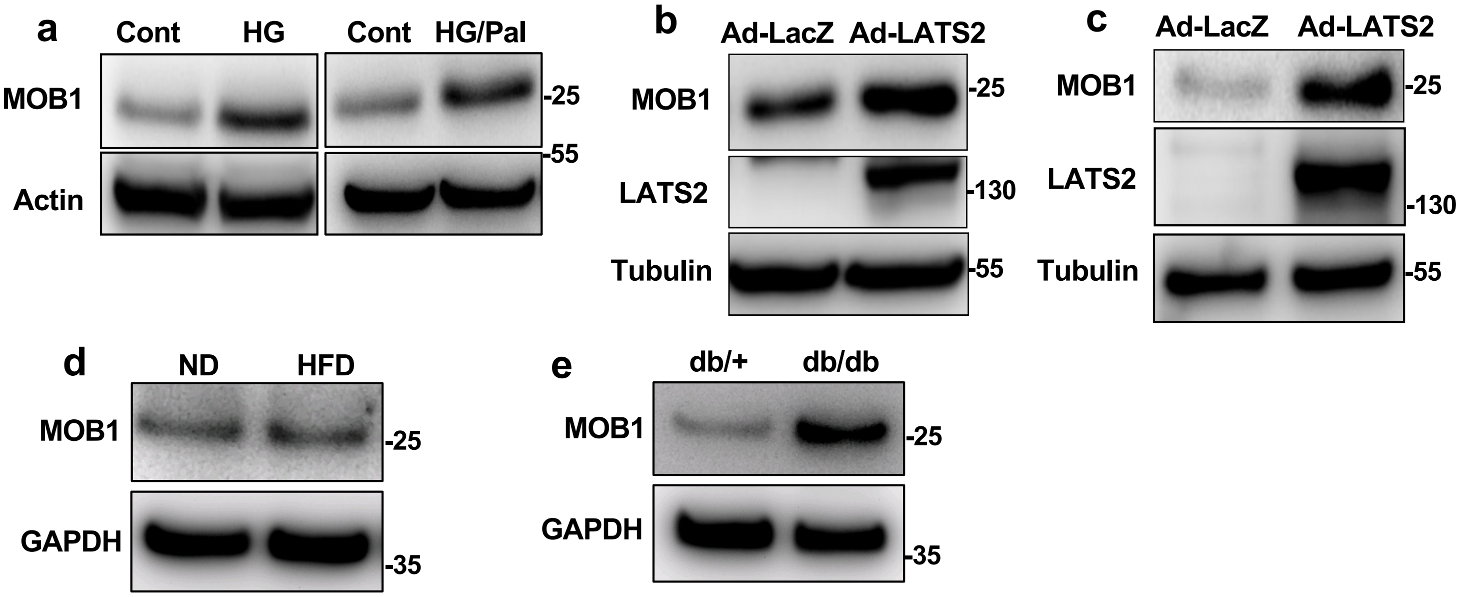

Supplementary Figure 1. MOB1 is upregulated by diabetogenic conditions and LATS2. Representative Western blots **(a)** of INS-1E cells treated with 22.2 mM glucose (HG) alone or with 0.5 mM palmitate (HG/Pal) for 48h, **(b,c)** of INS-1E cells **(b)** and human islets **(c)** transduced with LacZ control or LATS2 adenoviruses for 48 h, **(d,e)** of isolated islets from (d) HFD-treated C57BL/6J mice for 16 weeks or (e) from obese diabetic leptin receptor-deficient db/db mice and their corresponding controls cultured overnight and transfected with the N-luc-YAP15-S127 plasmid for 24 hours. GAPDH blots in d,e are from same experiment of Figure 1f,g, respectively. a,c-e: n=3 independent experiments; b: n=3 different human islets isolations.

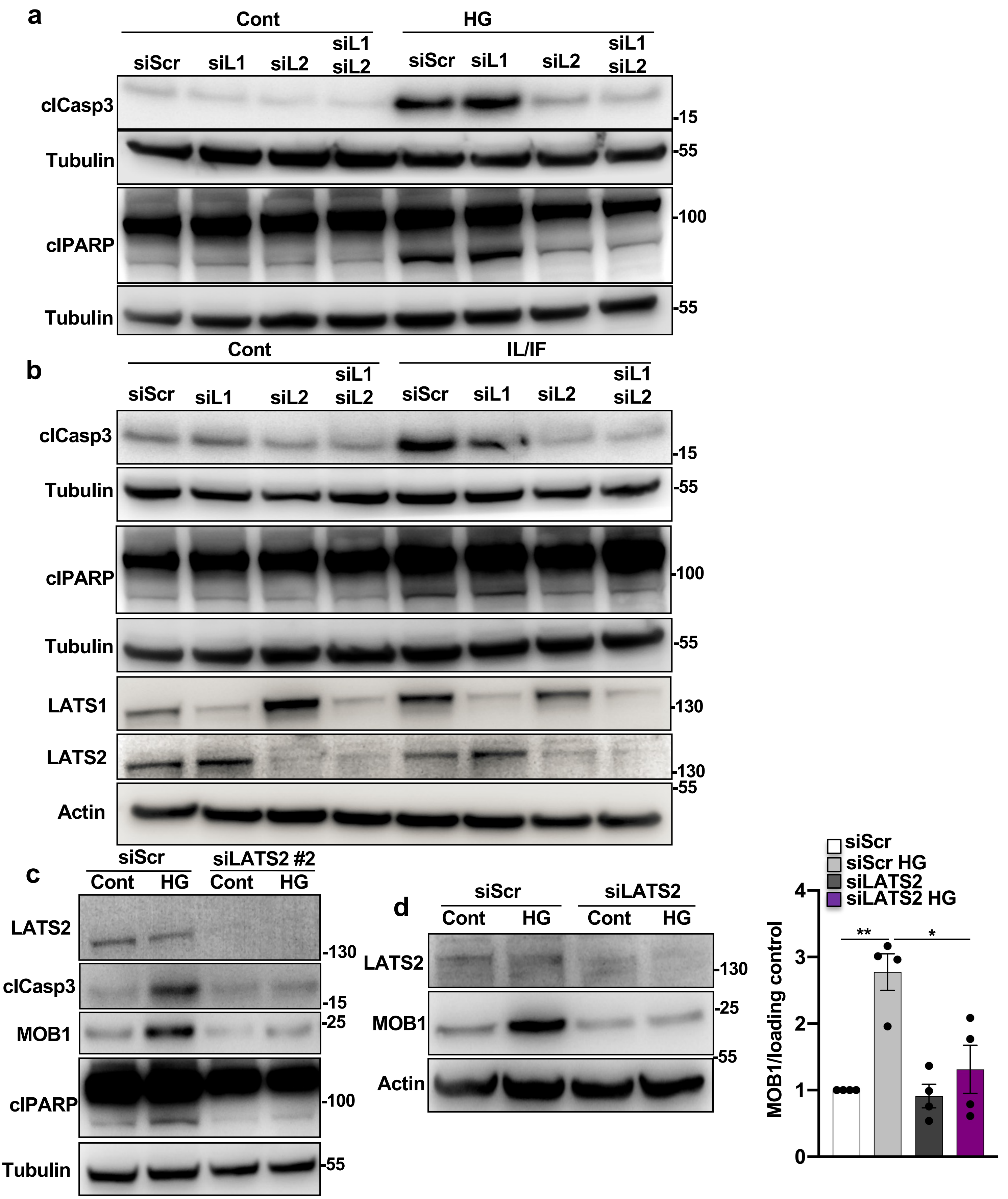

Supplementary Figure 2. LATS2 but not LATS1 knockdown protects from β-cell apoptosis. (a,b) Representative Western blots of INS-1E cells transfected with LATS1 and/or LATS2 siRNA or control siScr and treated with **(a)** 22.2 mM glucose (HG) or **(b)** 2 ng/mL IL1β plus 1000U/mL IFNγ (IL/IF) for 48 h, **(c)** of INS-1E cells transfected with LATS2 siRNA (second pool #2) or control siScr and treated with the 22.2 mM glucose (HG) for 48 h (a-c: n=1 experiment), **(d)** of INS-1E cells transfected with LATS2 siRNA or control siScr and treated with 22.2 mM glucose (HG) for 48 h with the respective pooled quantitative densitometry analysis (right panel; n=4 independent experiments). Data are expressed as means ± SEM. *p<0.05, **p<0.001; by two-tailed Student's *t*-test.

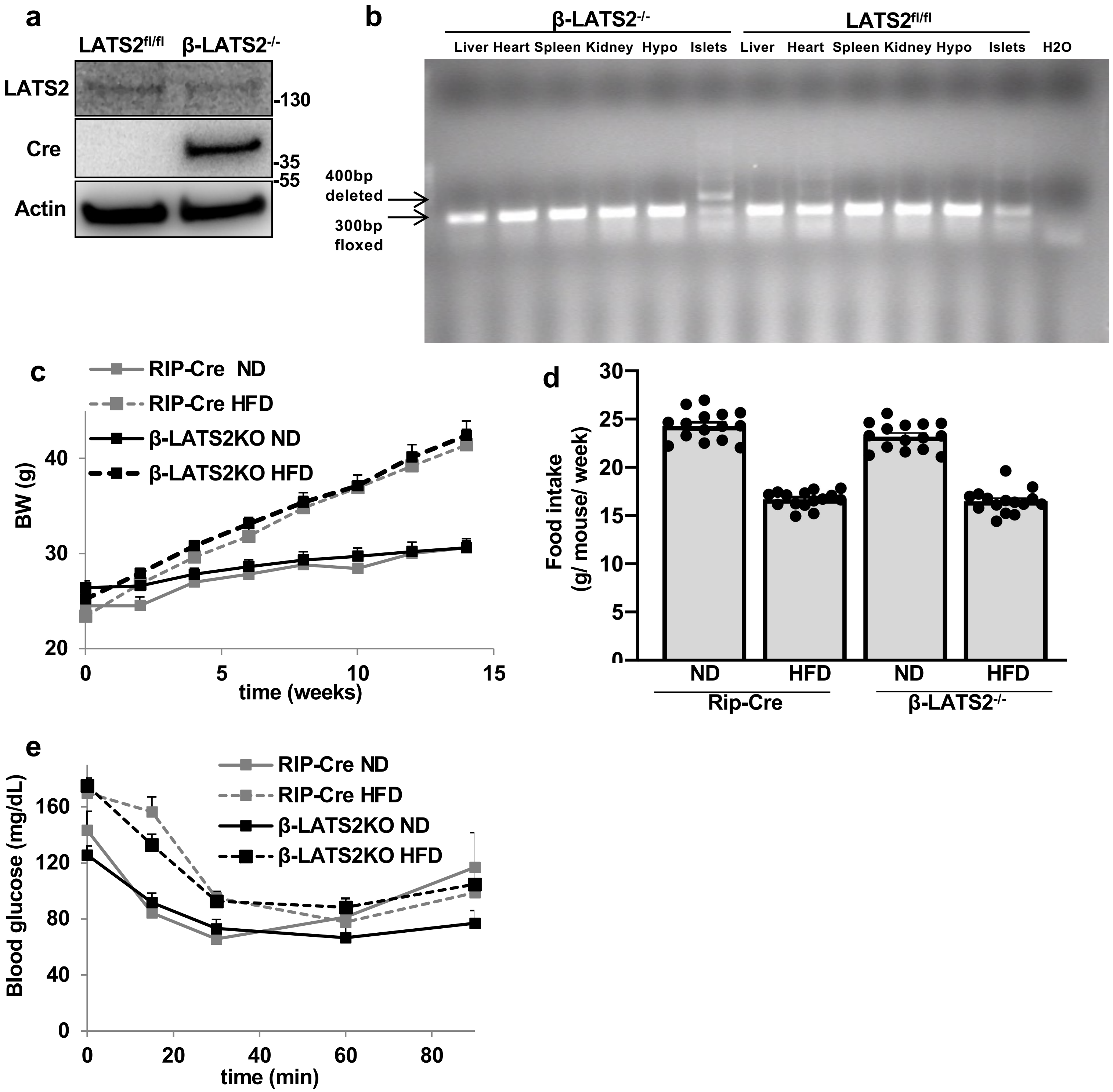

Supplementary Figure 3. Characterization of β-cell specific LATS2 knockout mice (β-LATS2$^{-/-}$). **(a)** Representative Western blot analysis of protein lysates from islets of β-LATS2$^{-/-}$ and LATS2$^{fl/fl}$ control mice (n=3 independent experiments). **(b)** PCR analysis of Cre-mediated LATS2 gene deletion in genomic DNA isolated from liver, heart, spleen, kidney, hypothalamus and pancreatic islets of β-LATS2$^{-/-}$ and LATS2$^{fl/fl}$ control mice (n=3 mice/group). **(c-e)** β-LATS2$^{-/-}$ and Rip-Cre control mice were fed a normal (ND) or high fat/ high sucrose diet ("Surwit"; HFD) for 17 weeks. **(c)** Body weight is expressed as means ± SEM from Rip-Cre ND n=8; Rip-Cre HFD n=19; β-LATS2$^{-/-}$ ND n=10; β-LATS2$^{-/-}$ HFD n=16 mice. **(d)** Average weekly food intake/mouse (average of n=15 weeks). **(e)** Intraperitoneal insulin tolerance test (ipITT) with 0.75IU/kg BW insulin. Data are expressed as means ± SEM from Rip-Cre ND n=7; Rip-Cre HFD n=17; β-LATS2$^{-/-}$ ND n=10; β-LATS2$^{-/-}$ HFD n=13 mice.

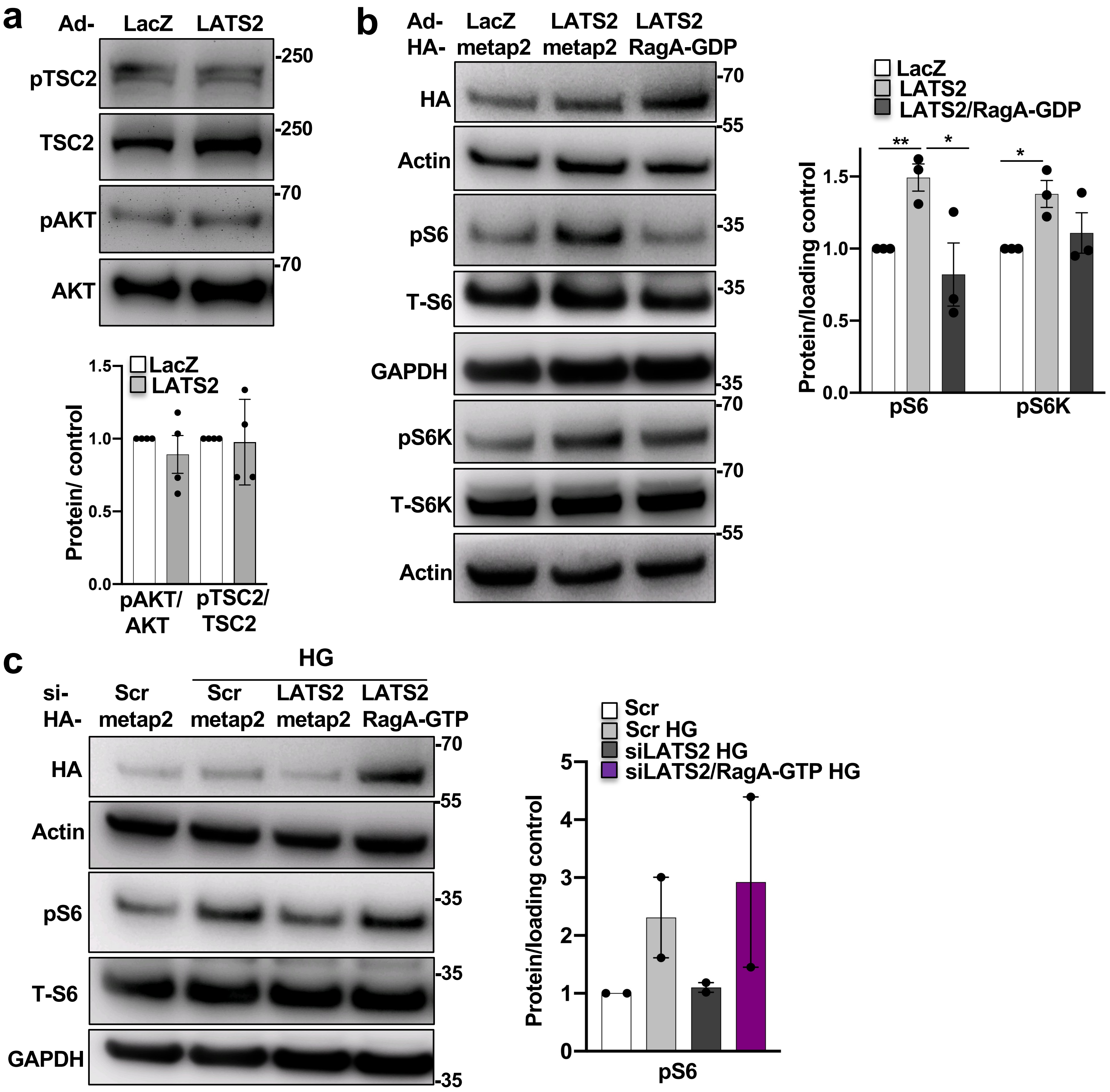

Supplementary Figure 4. LATS2-mTORC1 signaling axis. Representative Western blots and respective pooled quantitative densitometry analysis of **(a)** INS-1E cells transduced with LacZ control or LATS2 adenoviruses for 48 h (n=4 independent experiments), **(b)** of INS-1E cells transfected with dominant negative GDP bound RagA (HA-RagA-GDP; RagA-T21L) or metap2 (control) and then transduced with Ad-LacZ or Ad-LATS2 for 48 h (n=3 independent experiments), **(c)** of INS-1E cells transfected with siLATS2 or control siScr and constitutively active GTP bound RagA (HA-RagA-GTP; RagA-Q66L) or control metap2 and then treated with 22.2 mM glucose (HG) for 24 h (n=2 independent experiments). Data are expressed as means ± SEM. *p<0.05, **p<0.01; by two-tailed Student's *t*-tests.

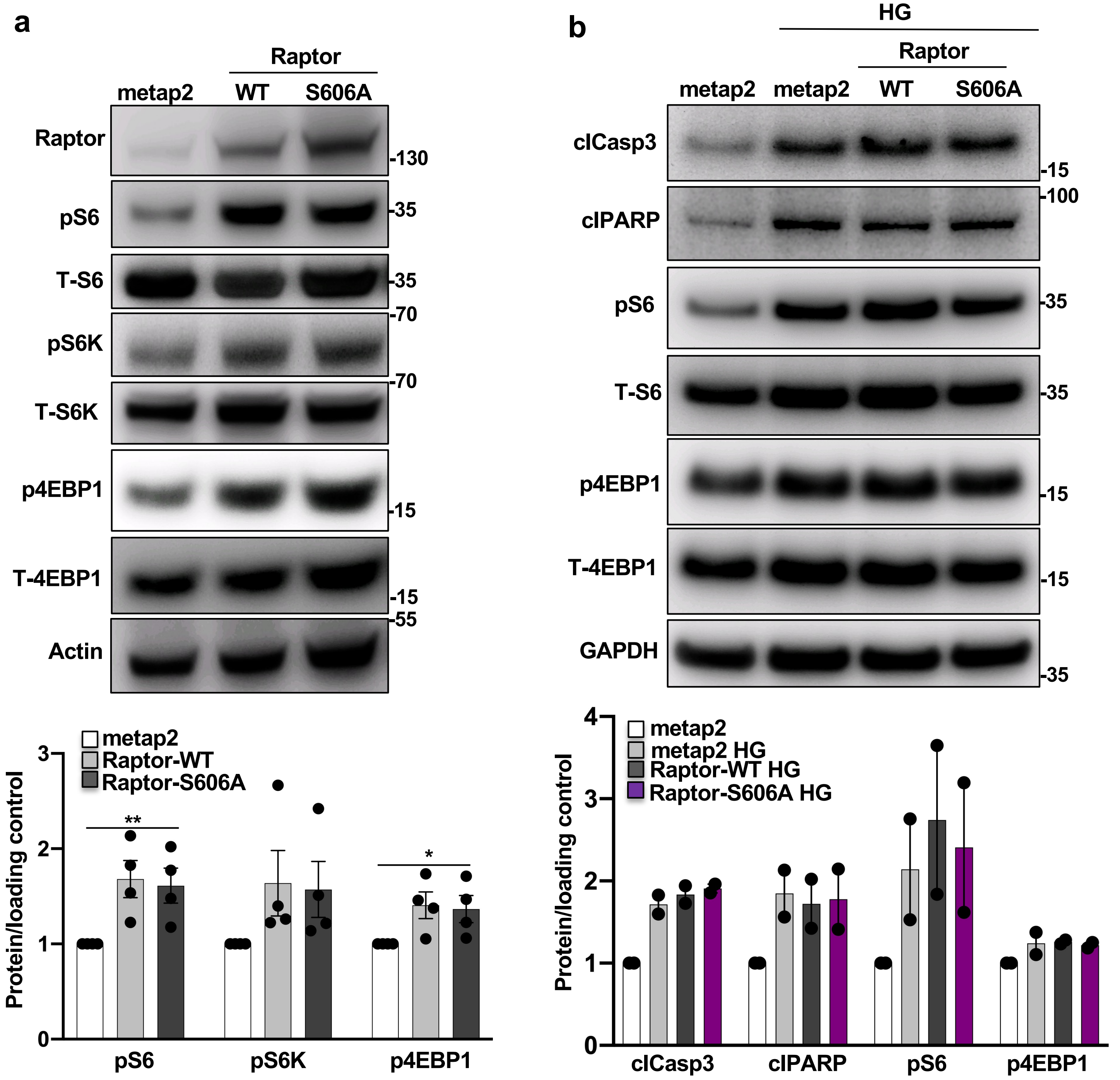

Supplementary Figure 5. Raptor-S606A mutant does not regulate mTORC1 and apoptosis in the β-cells. Representative Western blots (upper panels) and pooled quantitative densitometry analysis (lower panels) of **(a)** INS-1E cells transfected with Raptor-WT or Raptor-S606A mutant or control metap2 for 48 h (n=4 independent experiments), **(b)** of INS-1E cells transfected with Raptor-WT, Raptor-S606A mutant or control metap2 and then treated with 22.2 mM glucose (HG) for 24 h (n=2 independent experiments). Data are expressed as means ± SEM. *p<0.05, **p<0.01; by two-tailed Student's *t*-test.

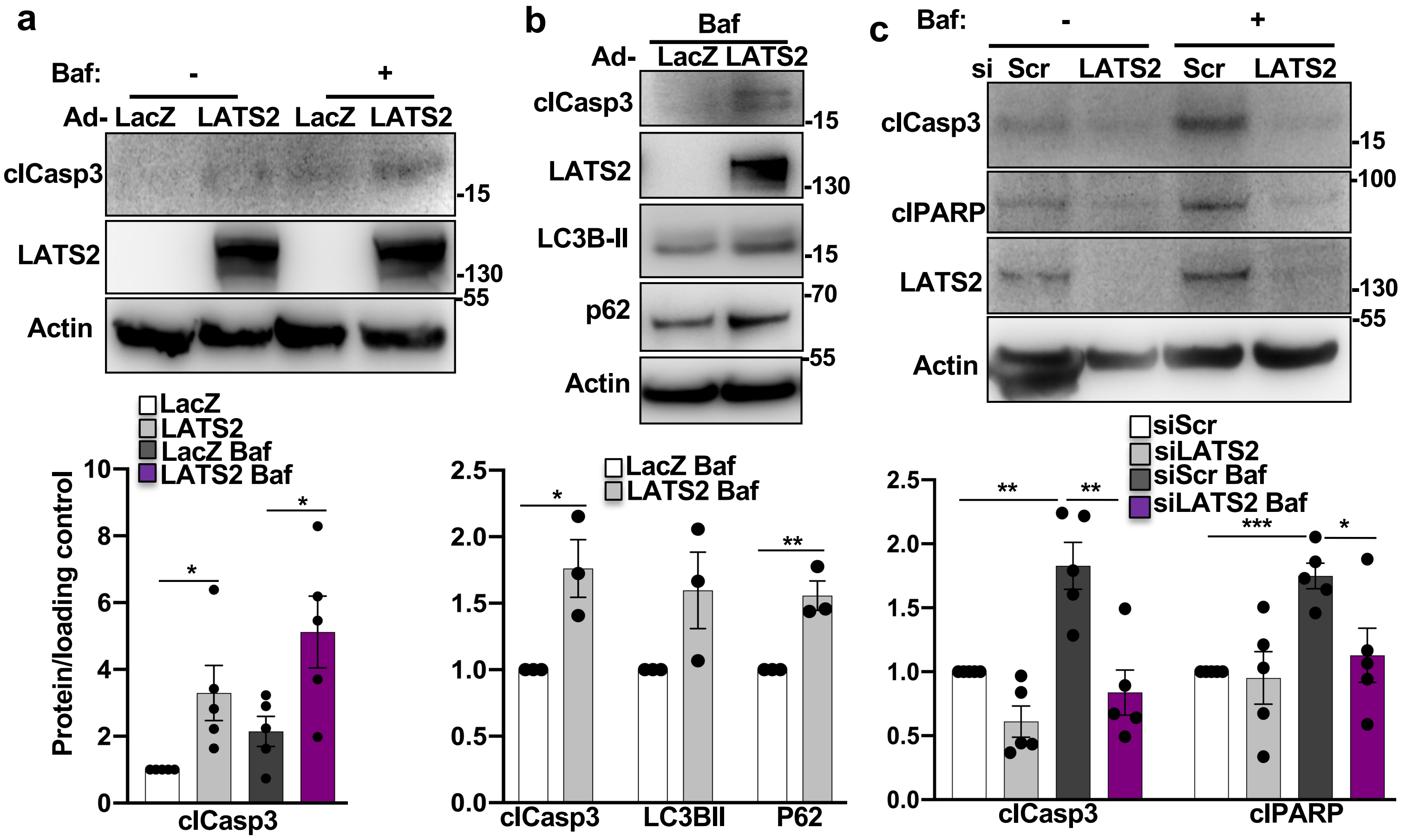

Supplementary Figure 6. LATS2-autophagy crosstalk. Representative Western blots and pooled quantitative densitometry analysis (lower panels) of INS-1E cells **(a)** and human islets **(b)** transduced with LacZ control or LATS2 adenoviruses and then treated with 20 nM Bafilomycin A1 (Baf) for 4h (n=5,3 independent experiments respectively for INS-1E and human islets), **(c)** of INS-1E cells transfected with siLATS2 or siScr and then treated with Bafilomycin A1 (n=5 independent experiments). Data are expressed as means ± SEM. *p<0.05, **p<0.01, ***p<0.001; by two-tailed Student's *t*-test.

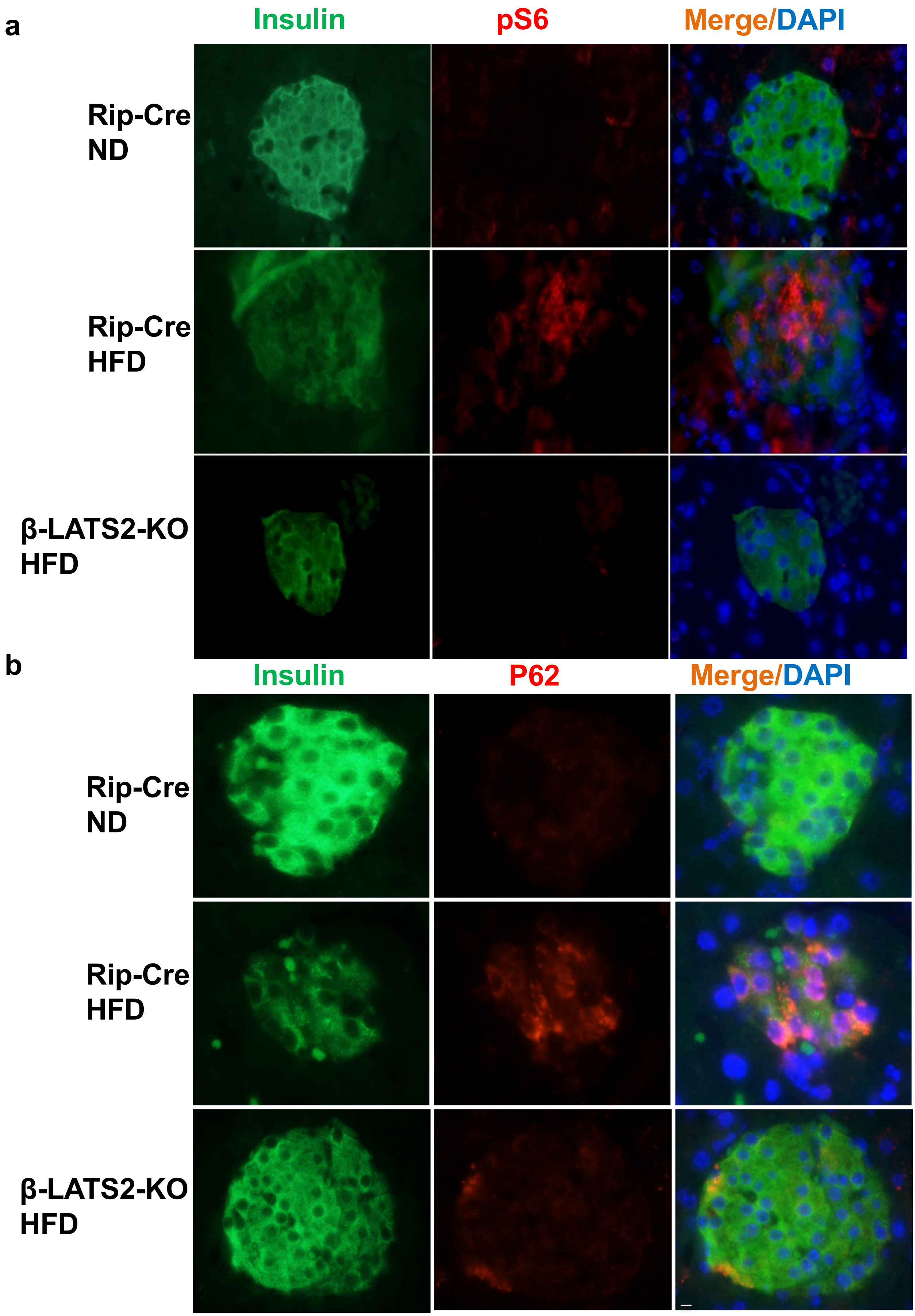

Supplementary Figure 7. LATS2 regulates mTORC1 and autophagy in HFD induced diabetic mice. Representative triple-stainings for phospho-S6 (pS6; red, a), or p62 (red, b), insulin (green) and DAPI (blue) are shown from pancreatic sections obtained from β-LATS2$^{-/-}$ and Rip-Cre control mice fed a normal (ND) or high fat/ high sucrose diet ("Surwit"; HFD) for 17 weeks. Scale bar depicts 10μm.

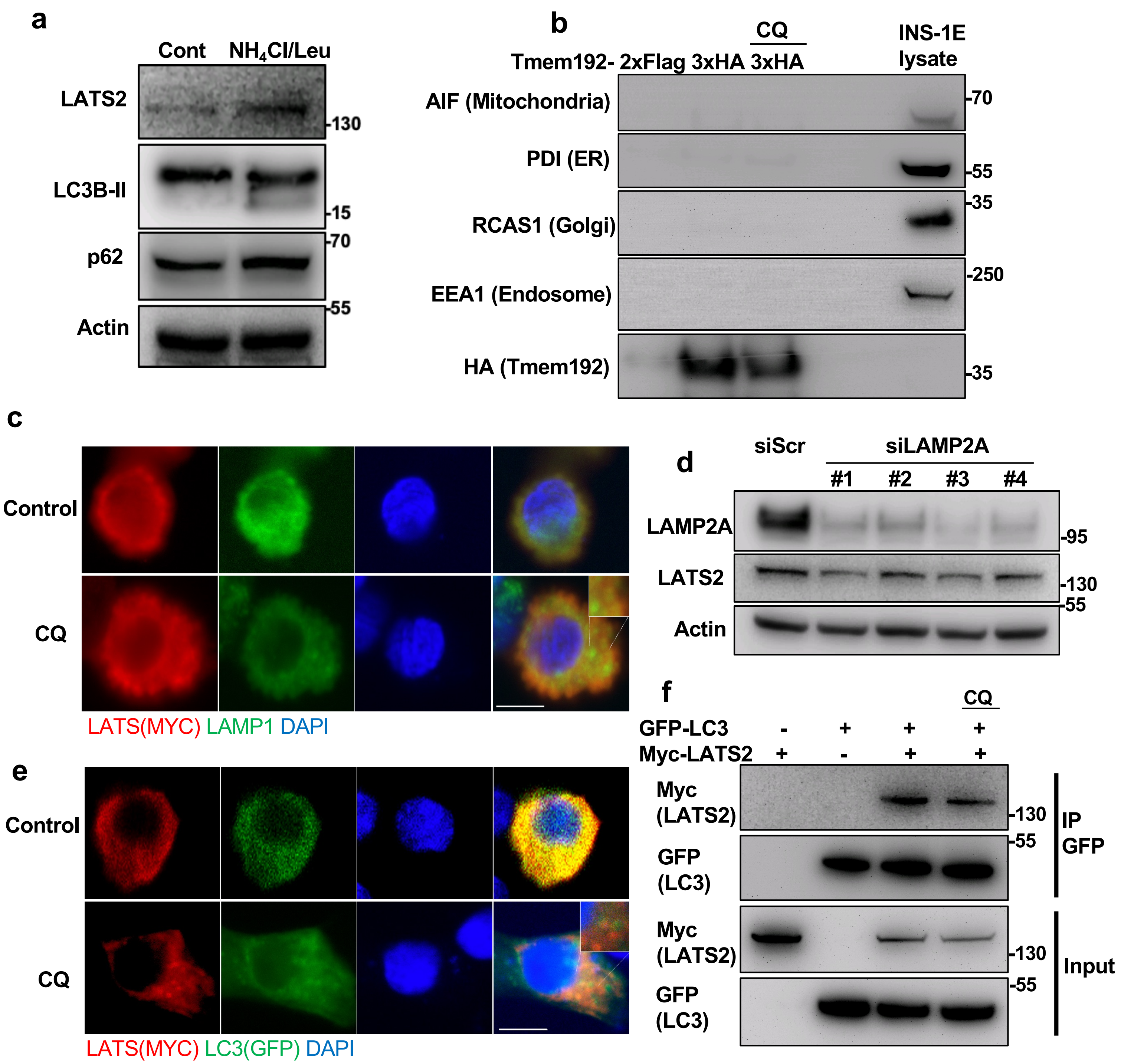

Supplementary Figure 8. LATS2-autophagy crosstalk. (a) Representative Western blot of INS-1E cells treated with a cocktail of 100 μM Leupeptin and 10 mM NH_4Cl for 12h. (b) INS-1E cells were co-transfected with LATS2-Myc and Tmem192-3xHA or Tmem192-2xFlag plasmids for 48h. One set of cells were treated with 50μM CQ for last 4h. Representative Western blot of lysosomes isolated from INS-1E cells together with INS-1E total lysate is shown. (c) Representative confocal microscopy-acquired triple-stainings for Myc-LATS2 (Myc; red) or LAMP1 (green) and DAPI (blue) are shown from human insulinoma CM β-cells left untreated or treated with CQ (100μM CQ for the last 6h). (d) Representative Western blot of INS-1E cells transfected with with siScr or LAMP2A for 72h. (e) Representative confocal microscopy-acquired triple-stainings for Myc-LATS2 (Myc; red) or LC3-GFP (GFP; green) and DAPI (blue) are shown from INS-1E cells left untreated or treated with CQ (100μM CQ for the last 6h). (f) INS-1E cells were transfected GFP-LC3 and /or LATS2-Myc plasmids for 48h. One set of cells were treated with 50μM CQ for last 4h. Representative Western blot of immunoprecipitation using anti-GFP magnetic beads is shown. Scale bar depicts 10μm.

a

Motif	Start	End	Pattern	PSSM Score
xLIR	666	671	SMFVKI	12
WxxL	80	85	IRYSLL	4
WxxL	181	186	ASYHQL	7
WxxL	214	219	YLFPGV	-3
WxxL	284	289	GGYASL	8
WxxL	653	658	SNYNRL	7
WxxL	677	682	GAFGEV	3
WxxL	840	845	DLWDDV	16
WxxL	888	893	KGYTQL	10
WxxL	894	899	CDWWSV	13
WxxL	902	907	ILFEML	7
WxxL	971	976	PFFSAI	3
WxxL	1000	1005	SNFDPV	10
WxxL	1020	1025	KAWDTL	16

b

```
>NP_055387.2 serine/threonine-protein kinase LATS2 [Homo sapiens]
MRPKTFPATTYSGNSRQRLQEIREGLKQPSKSSVQGLPAGPNSDTSLDAKVLGSKDATRQQQQMRATPKF
GPYQKALREIRYSLLPFANESGTSAAAEVNRQMLQELVNAGCDQEMAGRALKQTGSRSIEAALEYISKMG
YLDPRNEQIVRVIKQTSPGKGLMPTPVTRRPSFEGTGDSFASYHQLSGTPYEGPSFGADGPTALEEMPRP
YVDYLFPGVGPHGPGHQHQHPPKGYGASVEAAGAHFPLQGAHYGRPHLLVPGEPLGYGVQRSPSFQSKTP
PETGGYASLPTKGQGGPPGAGLAFPPPAAGLYVPHPHHKQAGPAAHQLHVLGSRSQVFASDSPPQSLLTP
SRNSLNVDLYELGSTSVQQWPAATLARRDSLQKPGLEAPPRAHVAFRPDCPVPSRTNSFNSHQPRPGPPG
KAEPSLPAPNTVTAVTAAHILHPVKSVRVLRPEPQTAVGPSHPAWVPAPAPAPAPAPAPAAEGLDAKEEH
ALALGGAGAFPLDVEYGGPDRRCPPPPYPKHLLLRSKSEQYDLDSLCAGMEQSLRAGPNEPEGGDKSRKS
AKGDKGGKDKKQIQTSPVPVRKNSRDEEKRESRIKSYSPYAFKFFMEQHVENVIKTYQQKVNRRLQLEQE
MAKAGLCEAEQEQMRKILYQKESNYNRLKRAKMDKSMFVKIKTLGIGAFGEVCLACKVDTHALYAMKTLR
KKDVLNRNQVAHVKAERDILAEADNEWVVKLYYSFQDKDSLYFVMDYIPGGDMMSLLIRMEVFPEHLARF
YIAELTLAIESVHKMGFIHRDIKPDNILIDLDGHIKLTDFGLCTGFRWTHNSKYYQKGSHVRQDSMEPSD
LWDDVSNCRCGDRLKTLEQRARKQHQRCLAHSLVGTPNYIAPEVLLRKGYTQLCDWWSVGVILFEMLVGQ
PPFLAPTPTETQLKVINWENTLHIPAQVKLSPEARDLITKLCCSADHRLGRNGADDLKAHPFFSAIDFSS
DIRKQPAPYVPTISHPMDTSNFDPVDEESPWNDASEGSTKAWDTLTSPNNKHPEHAFYEFTFRRFFDDNG
YPFRCPKPSGAEASQAESSDLESSDLVDQTEGCQPVYV
```

Supplementary Figure 9. Identification of LIR motifs in human LATS2 protein sequence. (a) Screenshot of sequences, position and PSSM scores of the several putative LIR and xLIR motifs for human LATS2 protein obtained from the iLIR database (https://ilir.warwick.ac.uk). (b) xLIR (green) and LIR (yellow) motifs are highlighted in FASTA sequence of human LATS2 protein.
